# Impaired Nuclear Export of the Ribonucleoprotein Complex and Virus-Induced Cytotoxicity Combine to Restrict Propagation of the A/Duck/Malaysia/02/2001 (H9N2) Virus in Human Airway Cells

**DOI:** 10.3390/cells9020355

**Published:** 2020-02-03

**Authors:** Sriram Kumar, Dawn Yeo, Nisha Harur Muralidharan, Soak Kuan Lai, Cathlyn Tong, Boon Huan Tan, Richard J. Sugrue

**Affiliations:** 1 School of Biological Sciences, Nanyang Technological University, 60 Nanyang Drive, Singapore 637551, Singapore; sriram.k.2017.mtechbt@rajalakshmi.edu.in (S.K.); yeodawn@dso.org.sg (D.Y.); nishahmuralidharan@gmail.com (N.H.M.); SKLai@ntu.edu.sg (S.K.L.); cathlyn.tong@gmail.com (C.T.); 2 Detection and Diagnostics Laboratory, DSO National Laboratories, 27 Medical Drive, Singapore 117510, Singapore; tboonhuan@gmail.com

**Keywords:** avian influenza virus, H9N2 virus, nucleoprotein, ribonucleoprotein (RNP) complex, PA protein, lamin A/C

## Abstract

In humans, (A549) cells impaired H9N2 virus nuclear export of the ribonucleoprotein (RNP) complex contrasted with the early and efficient nuclear export of the H1N1/WSN and pH1N1 virus RNP complexes. Although nuclear export of the RNP complex occurred via the nuclear pore complex, H9N2 virus infection also induced modifications in the nuclear envelope and induced cell cytotoxicity. Reduced PA protein levels in H9N2 virus-infected A549 cells occurred, and this phenomenon was independent of virus infection. Silencing the H1N1/WSN PA protein expression leads to impaired nuclear export of RNP complexes, suggesting that the impaired nuclear export of the H9N2 virus RNP complex may be one of the consequences of reduced PA protein levels. Early and efficient export of the RNP complex occurred in H9N2 virus-infected avian (CEF) cells, although structural changes in the nuclear envelope also occurred. Collectively our data suggest that a combination of delayed nuclear export and virus-induced cell cytotoxicity restricts H9N2 virus transmission in A549 cells. However, the early and efficient export of the RNP complex mitigated the effects of virus-induced cytotoxicity on H9N2 virus transmission in CEF cells. Our findings highlight the multi-factorial nature of host-adaptation of the polymerase proteins of avian influenza viruses in non-avian cell environments.

## 1. Introduction

The influenza A virus (IAV) polymerase complex consists of the PA, PB1 and PB2 polymerase proteins, and each protein possesses distinct biochemical properties (reviewed in [1]). The virus polymerase complex synthesizes new copies of genomic RNA (vRNA) and produces short methylated capped RNA primers from cellular mRNA that are used to prime virus mRNA synthesis. The PB2 protein contains a cap-binding domain that immobilizes the host-cell mRNA at the replication complex [2,3,4], while the methylated capped primers are generated by the endonuclease activity within the PA protein [5]. The PB1 protein contains the polymerase activity, the 5′ and 3′ binding sites for the vRNA, and the intercation domains for the PA and PB2 proteins [6,7,8,9,10]. The functional polymerase complex requires all the activities associated with the individual polymerase proteins, and the recent structural analysis of the entire polymerase complex has provided mechanistic insights on how these different activities are orchestrated [11,12,13]. The virus nucleoprotein (NP) coats the vRNA, and together with the polymerase complex forms a larger independent transcriptional unit called the ribonucleoprotein (RNP) complex [14,15,16,17,18]. The influenza virus nuclear export protein (NEP) interacts with the RNP complex in the nucleus and facilitates nuclear export of the newly formed RNP complex via the exportin 1/chromosome region maintenance 1 (crm1) export pathway [19]. The individual RNP complexes representing each of the eight virus gene segments are then exported to the site of virus assembly.

Although the capacity of influenza virus to infect different hosts is dependent upon several factors (reviewed in [20]), the biological properties of the virus polymerase complex is a major factor in the maintenance of the virus in a new host [21]. The activity of the polymerase complex in different hosts is dependent on the intrinsic properties of the polymerase complex (e.g., thermal stability) as well as interactions with specific host cell factors [22]. Specific amino acid sequence motifs that are associated with host adaptation have been identified within the different proteins that form the RNP complex. In some specific cases, biological functions associated with specific sequence motifs have been proposed [23,24], but in general, the role that these sequence motifs play in mediating species adaptation are generally poorly defined. Although there is a restriction in the sequence changes that can be accommodated in these proteins without adversely affecting their biological activity, a significant degree of sequence variation exists in these proteins among different avian influenza viruses. This sequence variation is likely to influence the interaction with specific host cell factors, and as a consequence the molecular processes that lead to host adaptation may be to some extent virus strain-specific. Therefore, a more complete picture of host adaptation of the influenza virus polymerase complex requires an improved understanding of host adaptation of the polymerase complex of avian viruses that are circulating in the natural environment. This includes those viruses that are not necessarily associated with human disease and which may be overlooked.

H9N2 influenza virus strains are widespread and responsible for disease outbreaks in poultry in many parts of the world, and in this context, it has an important impact on food security. Although H9N2 virus infection in humans has been reported [25,26], the disease symptoms are relatively mild [27]. Therefore compared with other highly pathogenic avian influenza viruses (e.g., H5N1) that cause severe disease in humans, infection by low pathogenic H9N2 viruses is often overshadowed in relation to public health control. The H9N2 virus can infect pigs and these are believed to be the intermediate species in many avian-to-human transmission events. Interestingly, a novel reassorted H7N9 virus was described in 2013 that was responsible for significant disease severity in humans [28,29]. The H7N9 virus contained six internal genes that originated from circulating H9N2 viruses [29,30,31] indicating that the H9N2 virus can contribute genes to reassorted viruses that cause more significant disease severity in humans. It is, therefore, important to understand the biological properties of H9N2 viruses that are circulating in the natural environment in order to determine their disease potential in humans.

We have previously described the biological properties of a low passaged tissue culture grown avian virus A/Duck/Malaysia/02/2001 (H9N2) that was isolated from live poultry during routine surveillance in Singapore [32]. The H9N2 virus could be readily cultured using embryonated eggs to yield virus preparations with high levels of infectivity, but it exhibited cell-specific variations in virus replication in tissue culture [33]. The A549 cell line is derived from the human airway tissue and is an established cell model with which to examine influenza virus replication. The H9N2 virus could efficiently infect A549 cells, but unlike the other circulating avian viruses in the study (e.g., H5N2), the H9N2 virus exhibited low replication rates in these cells. The MDCK cell line is an established culture system that is used to propagate human and avian influenza virus strains in tissue culture. The H9N2 virus could not be efficiently propagated in MDCK cells, and in standard plaque assay, it produced very small plaques in MDCK cells compared with other avian viruses e.g., H5N2 virus [33]. This suggested that in these cells H9N2 virus transmission was impaired. In contrast, significantly higher replication kinetics was observed in cells of avian origin infected with the H9N2 virus, consistent with its propagation in embryonated eggs. These observations suggested that the avian origin of the polymerase proteins in the H9N2 virus may be a factor that restricts its replication in cells derived from human or mammalian tissue, and may be an obstacle to propagation of the H9N2 virus in these cell types. In our earlier study we concluded that this restriction may be multi-factorial, and understanding the basic biological properties of the H9N2 virus replication complex could provide the basis for the poor recovery of the H9N2 virus using standard mammalian tissue culture systems. This information could also provide novel insights into the requirements of the H9N2 virus to adapt to a non-avian cell environment. We have therefore extended our earlier study to present a more detailed analysis of the H9N2 virus replication complex in human and avian cells using a cellular virology approach.

## 2. Materials and Methods

### 2.1. Cells and Viruses

The A549, HEK293, and CEF (UMNSAH/DF-1) cell lines were purchased from the American Type Culture Collection (ATCC) (Manassas, VA, USA). All cells were maintained in DMEM (Gibco), 10% FCS (Gibco), 1% antibiotic solution (penicillin-streptomycin 100 U/mL) at 37 °C with 5% CO_2_. The A/Duck/Malaysia/02/2001 (H9N2), pH1N1/2009 [32,33], A/ Umbrella Cockatoo/Singapore/F47/92 (H4N1), A/Fairy Blue Bird/Singapore/F92/94 (H7N1) and the A/WSN/1933 (H1N1/WSN) (VR-1520 (ATTC)) were used in this study. Virus stocks were prepared using 12 day-old specific pathogen-free embryonated chicken eggs. Cells were infected using the desired multiplicity of infection (MOI) in DMEM, 2% FCS, and 1% antibiotic solution at 37 °C with 5% CO_2_.

### 2.2. Specific Reagents

The anti-PA (mab9F5), and anti-PB2 (mab4G3) mouse monoclonal antibodies and the mouse anti-PB1 polyclonal antibody have been described previously [34]. The anti-NP (Chemicon, San Diago, CA, USA), anti-NEP and anti-M1 (ThermoFisher Scientific, Waltham, MA, USA), anti-actin and anti-FLAG (Sigma Aldrich, St louis, MO, USA), anti-lamin A/C and anti-lamin B1 (Santa Cruz, Dallas, Tx, USA), anti-Nuclear Pore Complex (NPC) (Abcam, Cambridge, UK) were purchased. The DAPI-dilactate and anti-mouse and anti-rabbit IgG conjugated to Alexa 488 and Alexa 555 respectively were purchased from Molecular Probes. The propidium iodide/RNAase solution was purchased from Cell Signaling Technology.

### 2.3. Immunoblotting Analysis of Whole-Cell Extracts

Cells were harvested and washed using PBS (4 °C) and extracted directly into Boiling Mix (final concentration: 1% SDS, 5% mercaptoethanol in 20 mM Tris/HCL, pH 7.5) and heated at 95 °C for 4 min. The cell extracts were clarified by centrifugation (13,000× *g* for 2 min) and the proteins separated by SDS-PAGE and transferred by Western blotting onto nitrocellulose membranes. The results obtained from the immunoblotting analysis were quantified using ImageJ (ver IJ1.46r). In this case, protein bands to be quantified were delineated and the density determined. This was compared with the background intensity in control blank lanes.

### 2.4. Nuclei Preparation

This was performed as described previously [35]. Briefly, cells were suspended in solution 1 (320mM sucrose, 2mM MgCl_2_, 1mM NaCl, 1mM potassium phosphate, pH 6.8) at 2 × 10^6^ cells/mL at 4 °C and centrifuged (1000× *g* for 6 min). The cell pellet was suspended in solution 2 (10 mM NaCl, 1mM potassium phosphate, pH 6.8) for 15 min and the cells recovered by centrifugation (800× *g*; 10 min). The cell pellet was suspended in solution 3 (320 mM sucrose, 1 mM MgCl_2_, 0.3% (*v*/*v*) triton X-100, 1 mM potassium phosphate, pH 6.3) at 4 × 10^6^ cells/mL at 4 °C in a Dounce homogenizer and after 10 strokes of the homogenizer the homogenate was centrifuged (800× *g*; 10 min). The resulting nuclear pellet was suspended in solution 3 and the Dounce homogenizer step repeated. The final nuclear pellet was analyzed further.

### 2.5. Construction of Recombinant Expression Vectors

This was performed using standard protocols. Briefly, total RNA was extracted from H9N2, pH1N1/2009 and H1N1/WSN virus-infected allantoic fluid using the RNeasy Kit (Qiagen, Hilden, Germany) and reversed transcribed using SuperScript II RT (Invitrogen) with a Uni12 primer 5′AGCAAAAGCAGG3′ [36]. Using the High Fidelity PCR system (Invitrogen), each gene segment was PCR-amplified with gene-specific primers as described [36] (Table S1a). The PCR product for each gene was purified using QIAquick Gel Extraction Kit (Qiagen) and cloned into pCR2.1-TOPO or pCR4-TOPO (Invitrogen, Carlsbad, CA, USA) and sequenced. The full-length virus genes were amplified using the High Fidelity PCR system (Invitrogen) using gene-specific primers with suitable restriction enzymes. The C-terminal FLAG-tagged PA proteins were cloned using a reverse primer containing a FLAG-tag sequence. The pCAGGS plasmid and the amplified gene products were digested with their respective restriction enzymes (NEB), ligated using T4 DNA ligase (Roche, Basel, Switzerland), and then transformed into EZ chemical-competent cells (Qiagen). The orientation and sequence of each virus sequence in the expression plasmid was confirmed by sequencing in both directions. The Gaussia Luciferase (Luc) gene was subcloned from the vector pNEBR-XGLuc (NEB) into the pPOL1 plasmid to generate pPOL1-Luc. The Luc gene was in the negative sense and was flanked by the 5′ and 3′ untranslated regions (UTRs) of the NS gene of influenza A/H1N1/PR8 virus.

### 2.6. Polymerase Reporter Assay

A total of 1μg DNA (200 ng per construct) was transfected into 293T cells using TransIT-LT1 transfection agent (MirusBio, Madison, WI, USA) according to the manufacturer’s recommendations. As a negative control, pPOL1-Luc plasmid alone was used to transfect into the cells. The assay was performed at 37 °C. Supernatants of transfected cells were harvested at fixed time points post-transfection and luciferase activities were measured using a Gaussia Luciferase Assay Kit (NEB), and the signals were read using a Fluoroskan Ascent FL (ThermoFisher Scientific, Waltham, MA, USA).

### 2.7. Immunofluorescence Microscopy

Cells seeded onto a coverslip were infected with the virus using an MOI of 5. At specific post-infection time, cells were fixed with 4% paraformaldehyde (PFA) for 20 min at 25 °C and permeabilized with 0.1% (*v/v*) triton X-100 for 15 min at 4 °C. Briefly, cells were then labeled with the appropriate primary and secondary antibody combinations for 1 h each and mounted on slides using Fluorescence Mounting Medium (Dako). The stained cells were visualized with a Nikon Eclipse 80i Microscope and an Etiga 2000R camera attached, using appropriate machine settings. The images of immunofluorescence-stained cells were recorded using Q Capture Pro ver 5.0.1.26 (Q Imaging) with the same exposure time for each antibody stained virus and cell combination. The image intensity of individual sets of cells (n = 100) was measured using ImageJ (ver IJ1.46r), and statistical analysis of the fluorescence measurements was performed using the student T-test and pairwise analysis. In cases where stained cells were examined by confocal microscopy, this was performed using a Zeiss 710 confocal microscope with Airyscan using appropriate machine settings. The images were examined and processed using Zen ver 2.3 software.

### 2.8. Propidium Iodide (PI) Staining

Cells were treated with the PI staining solution following the manufacturer’s instruction. The cells were either stained directly or the cells were first PFA-fixed and then stained using the PI solution.

### 2.9. siRNA Design and Transfections

The siPA-H1N1 (5′-GCAAUUGAGGAGUGCCUGA3′) [37], siNP and siGFP (5′GGCUACGUCCAGGAGCGCAUU3′) were purchased from Dhamacon (Lafayette, CO, USA). siRNAs experiments were performed using a 100 nM siRNA concentration in Lipofectamine 2000 as described previously [34]. Transfections were optimized using the siGlo probe Dhamacon (Lafayette, CO, USA).

### 2.10. Real-Time Quantitative PCR (qPCR)

This was performed as described previously [33]. Briefly, the total RNA was extracted from infected or transfected cells using RNeasy kit (Qiagen, Hilden, Germany) and reversed transcribed using SuperScript II RT (Invitrogen). Real-time qPCR was performed with the LightCycler 2.0 System, software version 5.32 (Roche) using specific primers (Table S1b) according to the protocol previously published [38].

### 2.11. TUNNEL (TUN) Staining

This was performed using the DeadEnd™ Fluorometric TUNNEL system (Promega) following the manufacturer’s instructions and as described previously [39]. Briefly, the cells were fixed with 4% PFA, permeabilized with 0.2% (*v/v*) triton X-100, and incubated with the equilibration buffer containing the nucleotide mix and the rTdT Enzyme for 1 h at 37 °C. The reaction was terminated using the stop solution and the stained cells mounted on a glass slide using mounting media and visualized using a Nikon Eclipse 80i Microscope with an Etiga 2000R camera attached.

## 3. Results

### 3.1. Expression of the H9N2 Virus Polymerase-Associated Proteins in A549 and CEF Cells

In this current study, we have compared the H9N2 virus with the established human-derived H1N1/WSN laboratory virus isolate and a human clinical pH1N1/471 virus isolate. The pH1N1/471 virus was isolated from a patient during the early stages of the 2009 influenza virus pandemic and contains the PA and PB2 proteins of avian origin [33,40]. In addition and when appropriate, we have also compared the H9N2 virus with H7N1 and H4N1 avian influenza viruses that were isolated during routine surveillance in the same geographical region.

The expression of the NP and polymerase proteins in A549 and CEF cells infected with the H1N1/WSN, pH1N1/471 (pH1N1) or H9N2 viruses were examined using two complementary approaches. In the first approach cell lysates were prepared and examined by immunoblotting with relevant antibodies. This allowed us to confirm the recognition and specificity of each antibody, and to assess the relative levels of each of the polymerase-associated proteins in the infected cells.

**Figure 1:**
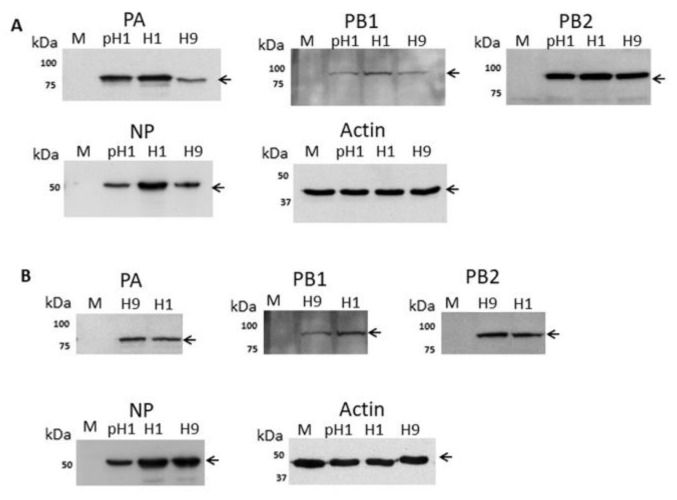
Analysis of the expression of the polymerase proteins in H1N1/WSN, pH1N1/471 and H9N2 influenza virus-infected cells by immunoblotting. (**A**) A549 and (**B**) CEF cells were mock-infected (M) or infected with either H1N1/WSN(H1), pH1N1/471 (pH1) or H9N2(H9) as indicated using a multiplicity of infection (MOI) of 5. At 20 h post-infection (hpi) the cells were harvested, extracted in boiling mix, and immunoblotted with anti-NP, anti-PB1, anti-PB2, anti-PA, and anti-actin (loading control). Protein bands are indicated (black arrow).

A549 cells were mock-infected or infected with the H1N1/WSN, pH1N1 or H9N2 viruses and at 20 h post-infection (hpi) cell lysates were immunoblotted using anti-NP, anti-PA, anti-PB1 and anti-PB2 (Figure 1A). Protein species of the expected sizes were detected and similar levels of the PB1 and PB2 proteins were detected. However, the expression level of the NP in H9N2 and pH1N1 virus-infected cells was approximately 60% of the NP level in H1N1/WSN virus-infected A549 cells. In addition, in the H9N2 virus-infected cells the PA protein expression level was approximately 30% of that detected in H1N1/WSN or pH1N1 virus-infected cells. In a similar manner CEF cells were mock-infected or infected with the H1N1/WSN or H9N2 viruses and at 20 hpi cell lysates were immunoblotted using anti-PA, anti-PB1, and anti-PB2. (Figure 1B). In both conditions, a comparable level of antibody staining was noted. In CEF cell lysates immunoblotted with anti-NP a comparable protein level was detected in H1N1/WSN or H9N2 virus-infected cells, although reduced NP levels were still detected in pH1N1 virus-infected CEF cells. These data suggested that in A549 cells there was a differential expression of the polymerase-associated proteins in a virus-specific manner.

In a second approach, antibody-stained infected cells were examined by immunofluorescence (IF) microscopy. Images were recorded for each virus and cell combination using identical camera settings, and the relative staining intensities of individually infected cells within the field of view were assessed and compared using densitometry. This allowed a more reliable method to directly assess the antibody staining intensity of individual anti-NP and anti-PA stained H1N1/WSN and H9N2 virus-infected A549 and CEF cells.

**Figure 2:**
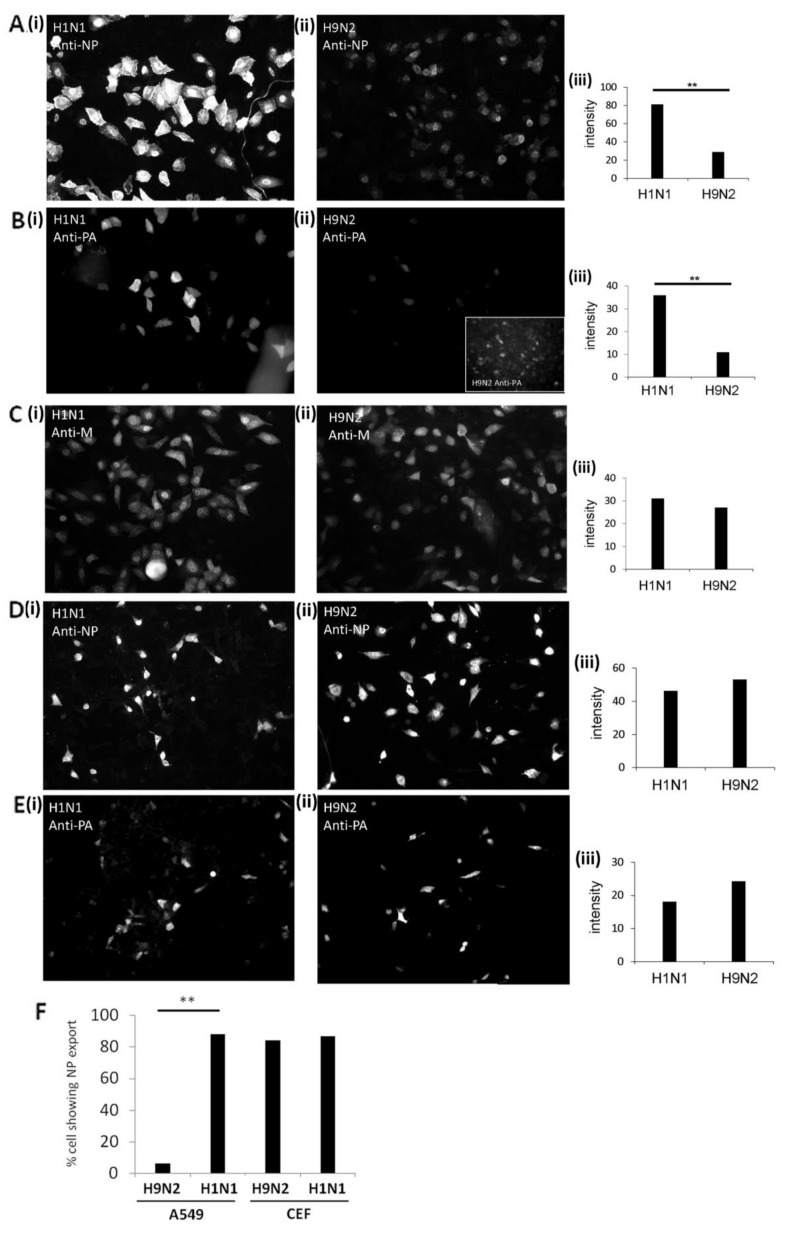
Image analysis of the expression of the polymerase proteins in H1N1/WSN, and H9N2 influenza virus-infected cells. (**A**–**C**) A549 and (**D**,**E**) CEF cells were infected with either (i) H1N1/WSN (H1N1) or (ii) H9N2 influenza virus using an MOI of 5 and at 20 h post-infection (hpi) the cells were stained using anti-NP (**A**,**D**), anti-PA (**B**, **E**) and anti-M (**C**) and imaged by fluorescence microscopy using identical camera settings (objective x20 magnification) for each antibody and cell combination. (**B**(ii)) inset shows anti-PA staining of H9N2 virus-infected cells using higher exposure times. (iii) The average fluorescence intensity of the individual stained cells obtained using the same camera settings was quantified using image J (n = 100). (**F**) The percentage of cells exhibiting high levels of nuclear export were also estimated for both the H1N1 and H9N2 viruses in A549 and CEF cells (i.e., prominent cytoplasmic anti-NP staining compared with detectable nuclear anti-NP staining). A representative analysis is presented, and n = 80. ** *p* < 0.005.

The NP and anti-PA staining levels in H9N2 virus-infected A549 cells were approximately 30% of that measured in the H1N1/WSN virus-infected cells (Figure 2A,B). In contrast, comparable staining intensities were recorded in anti-M stained H1N1/WSN and H9N2 virus-infected A549 cells (Figure 2C). The anti-M recognizes the M1 protein and serves as a control to assess the level of virus infection in the A549 cells. The imaging analysis indicated similar numbers of infected cells for both viruses and provided additional evidence that the reduced staining intensities with anti-NP and anti-PA were not due to a reduced number of H9N2 virus-infected cells. The image analysis of anti-NP and anti-PA stained CEF cells infected with H1N1/WSN and H9N2 viruses showed comparable anti-NP and anti-PA staining intensities for both viruses (Figure 2D,E). These data are consistent with differential expression of the H9N2 virus NP and PA protein in A549 cells, and confirmed that the differences in protein expression observed in A549 cells by immunoblotting analysis was not due to differences in the number of infected cells.

We used the imaging to estimate the level of RNP nuclear export in each virus and cell combination (Figure 2F). Approximately 95% of the H1N1/WSN virus-infected A549 cells exhibited a high level of cytoplasmic anti-NP staining, and was consistent with efficient nuclear export of the NP. In contrast, only 5–10% of H9N2 virus-infected cells showed high levels of cytoplasmic anti-NP staining, while greater than 90% of the cells showed enhanced anti-NP staining in the nucleus; consistent with impaired nuclear export of the NP. In CEF cells a prominent cytoplasmic anti-NP staining was noted for both viruses (Figure 2D), indicating that there was efficient nuclear export of the RNP complex in CEF cells that were infected with either virus.

### 3.2. Impaired Nuclear Export of the RNP Complex Occurs in H9N2 Virus-Infected A549 Cells

**Figure 3:**
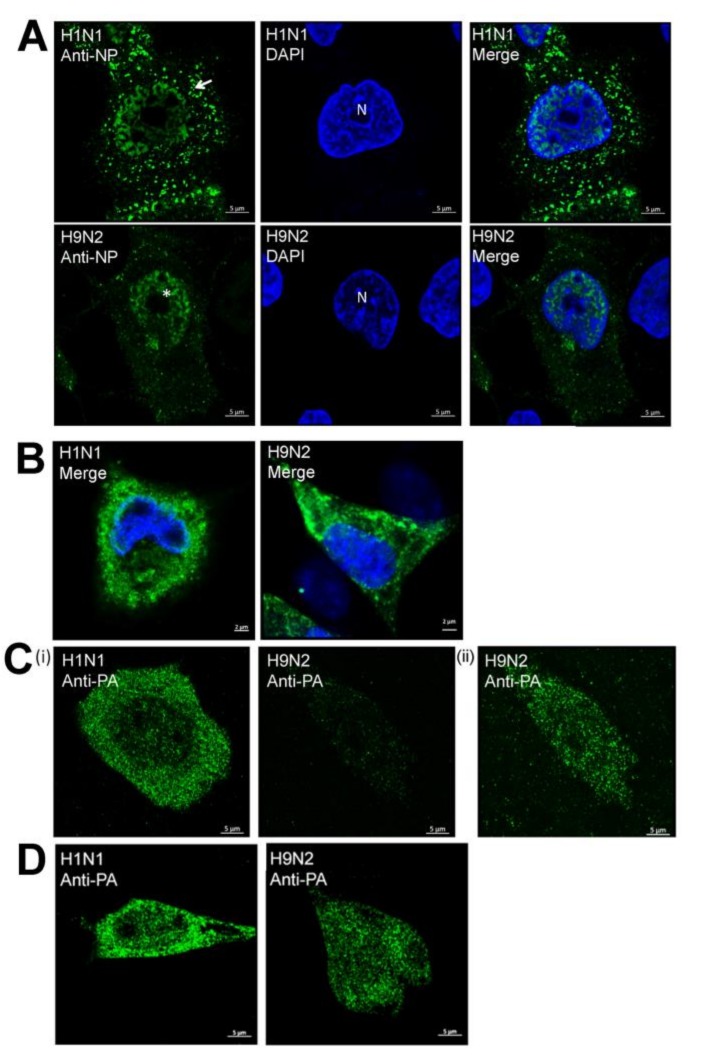
Analysis of the distribution of the NP and PA protein in H1N1/WSN, and H9N2 influenza virus-infected A549 and CEF cells. At 20 h post-infection (hpi) (**A**) A549 and (**B**) CEF cells infected with H1N1 and H9N2 viruses were co-stained using DAPI (blue) and anti-NP (green) and examined using confocal microscopy. The location of the nucleus (N), cytoplasmic NP staining in the H1N1 virus-infected cells (white arrow) and enhanced nuclear NP staining in H9N2 virus-infected cells (*) are highlighted. (**C**) A549 and (**D**) CEF cells infected with H1N1 and H9N2 viruses were co-stained using anti-PA (green) at 20 hpi and examined using confocal microscopy. In each plate, representative cells are shown, and in each cell and antibody staining combination identical machine settings were used. In (**C**(ii)) the same cell as in (**C**(i)) is viewed using higher laser energy to view the PA staining pattern.

A549 cells were infected with H1N1/WSN and H9N2 viruses and co-stained using anti-NP and DAPI and examined in greater detail using confocal microscopy (Figure 3A). This confirmed the cytoplasmic anti-NP staining in the H1N1/WSN virus-infected cells suggesting that efficient nuclear export of the RNP complex. In cells infected with the H9N2 virus, the NP staining was largely retained in the nucleus and was consistent with impaired nuclear export of the RNP complex. Examination of anti-NP and DAPI co-stained H1N1 or H9N2 virus-infected CEF cells by confocal microscopy revealed a prominent cytoplasmic anti-NP staining in each case (Figure 3B), indicating that efficient nuclear export of the RNP complex had occurred in CEF cells that were infected with either virus.

Imaging using confocal microscopy showed that in addition to the cytoplasmic anti-NP staining, the H1N1 virus-infected cells generally showed higher levels of anti-NP staining at the periphery of the DAPI-stained nucleus. This was consistent with the movement of the NP to the nuclear envelope and then into the cytoplasm i.e., efficient nuclear export of the RNP complex. In contrast, in H9N2 virus-infected cells the anti-NP staining was generally distributed uniformly across the nucleus, which was consistent with impaired nuclear export of the RNP complex in the H9N2 virus-infected cells. We also examined anti-PA stained A549 and CEF cells infected with H1N1/WSN and H9N2 viruses using confocal microscopy (Figure 3C,D). This confirmed the reduced levels of anti-PA staining in H9N2 virus-infected A549 cells when compared with H9N2 virus-infected CEF cells.

The histone H4 protein is an abundant protein found in chromatin, and co-staining the H1N1 and H9N2 virus-infected cells with anti-NP and anti-histone H4 (anti-H4) allowed us to determine the relative distribution of the RNP complexes and chromatin in the nucleus of infected cells (Supplementary Figure S1A). High levels of anti-NP staining at the periphery of the nucleus in the H1N1 virus-infected cells imaged using confocal microscopy were consistent with nuclear export of the RNP complex in these cells (Supplementary Figure S1B). In contrast, in H9N2 virus-infected cells the anti-NP staining was evenly distributed across the nucleus (Supplementary Figure S1C), and although co-localization between anti-NP and anti-H4 staining was not observed, the anti-NP staining appeared distributed in between the anti-H4 stained chromatin throughout the nucleus. This suggested that the RNP complexes might be retained spatially close to the chromosome within the nucleus of the H9N2 virus-infected cells.

**Figure 4:**
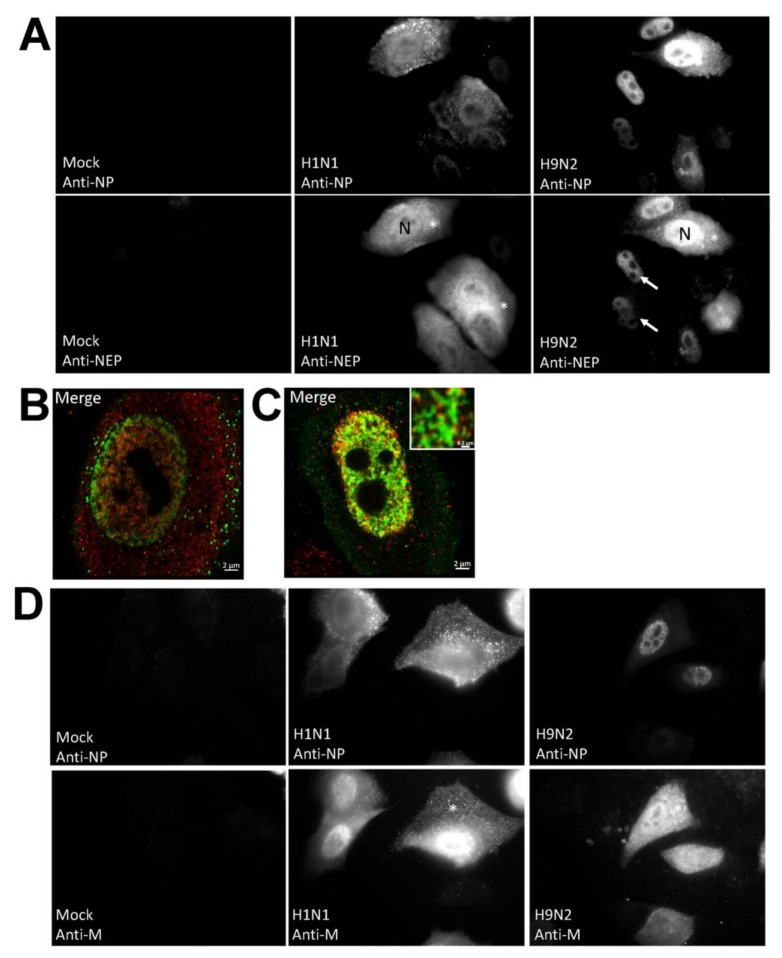
Distribution of the nuclear export protein (NEP) in H1N1/WSN, and H9N2 influenza virus-infected A549 cells. (**A**) A549 cells were mock-infected or infected with either H1N1/WSN(H1N1) or H9N2 using an MOI of 5 and at 20 hpi the cells were co-stained using anti-NP and anti-NEP. The cells were imaged by fluorescence microscopy using identical camera settings (objective ×100 magnification). The nucleus (N), nuclear (white arrow) and cytoplasmic (*) anti-NEP staining are highlighted. Individual (**B**) H1N1/WSN(H1N1) and (**C**) H9N2 virus-infected cells co-stained with anti-NP (green) and anti-NEP (red) were examined in greater detail using confocal microscopy. The merged image is shown in each case. Inset in (**C**) is an enlarged image showing the anti-NP and anti-NEP co-staining in the nucleus of H9N2 virus-infected cells. Bar = 0.5 μm. (**D**) A549 cells were mock-infected or infected with either H1N1/WSN(H1N1) or H9N2 were co-stained using anti-NP and anti-M and the cells imaged by fluorescence microscopy using identical camera settings (objective ×100 magnification). The cytoplasmic anti-M staining is highlighted (*).

Since the NEP is an integral part of the RNP complex that is destined for the nuclear exit we also examined the cellular distribution of the NEP (Figure 4A). A high level of cytoplasmic anti-NEP staining was detected in H1N1/WSN virus-infected cells and was consistent with the efficient export of the RNP complex. In the H9N2 virus-infected cells the anti-NP staining distribution correlated with anti-NEP staining, and in most cells staining with both antibodies was enriched in the nucleus. The correlation between the anti-NP and anti-NEP staining provided evidence that the anti-NP staining indicated the location of the RNP complex. It was noted that approximately 10% of the H9N2 virus-infected cells exhibited cytoplasmic anti-NP and anti-NEP staining, suggesting that nuclear export of the RNP complex in H9N2 virus-infected cells was impaired rather than being completely inhibited. A detailed analysis of the anti-NEP and anti-NP co-stained cells using confocal microscopy was performed (Figure 4B,C). This demonstrated that the anti-NEP staining in the nucleus appeared to be in close proximity to the anti-NP staining in both H1N1 and H9N2 virus-infected cells, although higher levels of nuclear-staining by each antibody was noted in the H9N2 virus-infected cells. While anti-NEP and anti-NP staining was detected in the cytoplasm in H1N1 virus-infected cells, co-staining was not apparent. In a similar analysis performed on anti-NP and anti-M co-stained H1N1/WSN and H9N2 virus-infected cells, the anti-M staining appeared in both the nucleus and cytoplasm (Figure 4D). Although the M1 protein is a major structural protein in virus particles, a small proportion of the M1 protein also associates with the RNP complex during nuclear export which accounts for the detection of the anti-M protein staining in the nucleus.

Examination of anti-NP stained-A549 cells infected with two other avian viruses (H4N1 and H7N1) isolated during routine surveillance was performed using IF microscopy (Supplementary Figure S2). Although both viruses contain avian signature sequences in the NEP, both viruses exhibited efficient nuclear export of the NP. This suggested that the impaired nuclear export of the H9N2 virus NP was not solely attributable to the avian origin of the virus, but may be an inherent property of the H9N2 virus. Similarly, image analysis of pH1N1 virus-infected A549 cells indicated reduced anti-NP staining intensities but high levels of cytoplasmic NP staining (Supplementary Figure S2A). This suggested that the low NP expression in the H9N2 virus-infected cells was not the direct cause of the impaired nuclear export.

### 3.3. Reduced Expression of the H9N2 Virus NP and PA Proteins in Human Cells is Independent of Virus Infection

**Figure 5:**
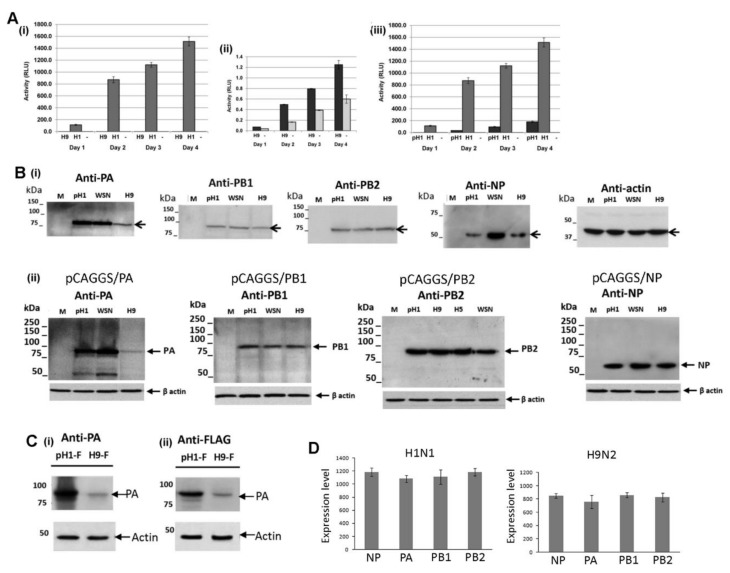
Expression of the recombinant H9N2 virus replication complex. (**A**) HEK293T cells were transfected with pCAGGS (M) and pPol1/luc (-) or co-transfected with pPol1/luc and pCAGGS/PA, pCAGGS/PB1, pCAGGS/PB2, pCAGGS/NP of the H1N1/WSN(H1), pH1N1/471 (pH1) or H9N2(H9) viruses. The polymerase activity was assessed by measuring the luciferase activity (RLU) in the tissue culture supernatant over four days. The respective polymerase activities of ((i) and (ii)) the H1N1/WSN(H1) and H9N2(H9) and (iii) the H1N1/WSN(H1) and pH1N1/471 (pH1) are shown. In each case luciferase activity (RLU) in cells transfected with pCAGGS (M) and pPol1/luc (-) is shown. (**B**) (i) After 24 hrs post-transfection (hptr) cells expressing the polymerase complex of each virus were examined by immunoblotting using anti-PA, anti-PB1, anti-PB2, and anti-NP. Protein bands corresponding in size to the respective full-length protein (black arrow) are indicated. Actin is the loading control and cells transfected with the parent vector pCAGGS is indicated (M). (ii) Cells were transfected with pCAGGS (M), pCAGGS/PA, pCAGGS/PB1, pCAGGS/PB2, pCAGGS/NP of the H1N1/WSN(H1), pH1N1/2009 or H9N2(H9) viruses. At 24 hptr the cells were examined by immunoblotting using anti-PA, anti-PB1, anti-PB2 and anti-NP respectively. Protein bands corresponding in size to the full-length polymerase proteins are indicated (black arrow), and in each case actin is the loading control. Also shown is expression of the recombinant H5N2 PB2 protein (H5). (**C**) Cells were transfected with pCAGGS/PA-FLAG of the pH1N1/471 (pH1-F) or H9N2 (H9-F) viruses and after 20 hptr the cells were immunoblotted using either (i) anti-PA or (ii) anti-FLAG. The location of the PA-FLAG protein is indicated (PA) and actin is the loading control. (**D**) HEK 293T cells were transfected with either pCAGGS/PA, pCAGGS/PB1, pCAGGS/PB2 or pCAGGS/NP of the H1N1/WSN (H1N1) and H9N2 virus and after 24 hptr the mRNA levels were quantified using PCR. Representative data from one experiment is shown.

It was not certain if the reduced expression of the H9N2 virus NP and PA proteins in A549 cells was a consequence of the host response to virus infection or if this was an intrinsic property of both virus proteins. This was addressed using an established mini-replicon system to reconstitute the recombinant H1N1/WSN, pH1N1 and H9N2 virus polymerase complexes in non-infected cells (Figure 5). The PB2, PB1, PA and NP genes for each virus were inserted into pCAGGS to generate pCAGGS/PB2, pCAGGS/PB1, pCAGGS/PA, and pCAGGS/NP. The pPOL1/luc is a reporter plasmid that contained the Gaussia luciferase (luc) gene which is flanked by the 3′and 5′ non-coding region (NCR) of segment-8. The five plasmids were co-transfected into HEK293T cells and the activity of the recombinant polymerase complex for each virus was measured. The H9N2 virus (Figure 5A(i),(ii)) and pH1N1 virus (Figure 5A (iii)) polymerase complexes exhibited less than 0.1% and 10% of the activity of the H1N1/WSN virus polymerase complex respectively, which was consistent with the measurement of polymerase activities in infected cells [33].

Cells expressing the recombinant replication complex derived from each virus were immunoblotted using anti-NP, anti-PA, anti-PB1, and anti-PB2, and in all cases, protein bands corresponding to the NP, PA, PB1 and PB2 proteins were detected (Figure 5B(i)). Reduced NP expression was detected in cells expressing the H9N2 and pH1N1 virus replication complexes, while similar NP expression levels for each of the three viruses were noted in cells singly transfected with corresponding pCAGGS/NP (Figure 5B(ii); Supplementary Figure S3A). This indicated that the reduced H9N2 and pH1N1 virus NP expression occurred when the NP was co-expressed with the other polymerase proteins.

Reduced H9N2 PA protein levels were observed in co-transfected cells (Figure 5B (i)) and in cells singly transfected with the H9N2 virus pCAGGS/PA (Figure 5B (ii)), Supplementary Figure S3B). The FLAG-epitope is a linear epitope that allows PA protein expression to be detected independently of anti-PA antibody recognition, and reduced H9N2 virus PA-FLAG protein expression levels were observed when immunoblotted with either anti-PA (Figure 5C(i)) or anti-FLAG (Figure 5C(ii)). This confirmed that the reduced H9N2 virus PA protein detection was not due to reduced anti-PA recognition i.e., reduced anti-PA binding. The mRNA levels were measured in cells singly transfected with the pCAGGS/PA, pCAGGS/PB1, pCAGGS/PB2 and pCAGGS/NP of the H1N1/WSN or H9N2 viruses (Figure 5D). Similar mRNA levels were detected in cells transfected with the plasmid set for each virus, providing evidence that the reduced H9N2 PA protein levels were not due to reduced PA mRNA expression levels.

### 3.4. Reduced Expression of the H9N2 NP and PA Proteins is not Mitigated by Co-Expression with the Other H1N1/WSN Polymerase Proteins

**Figure 6:**
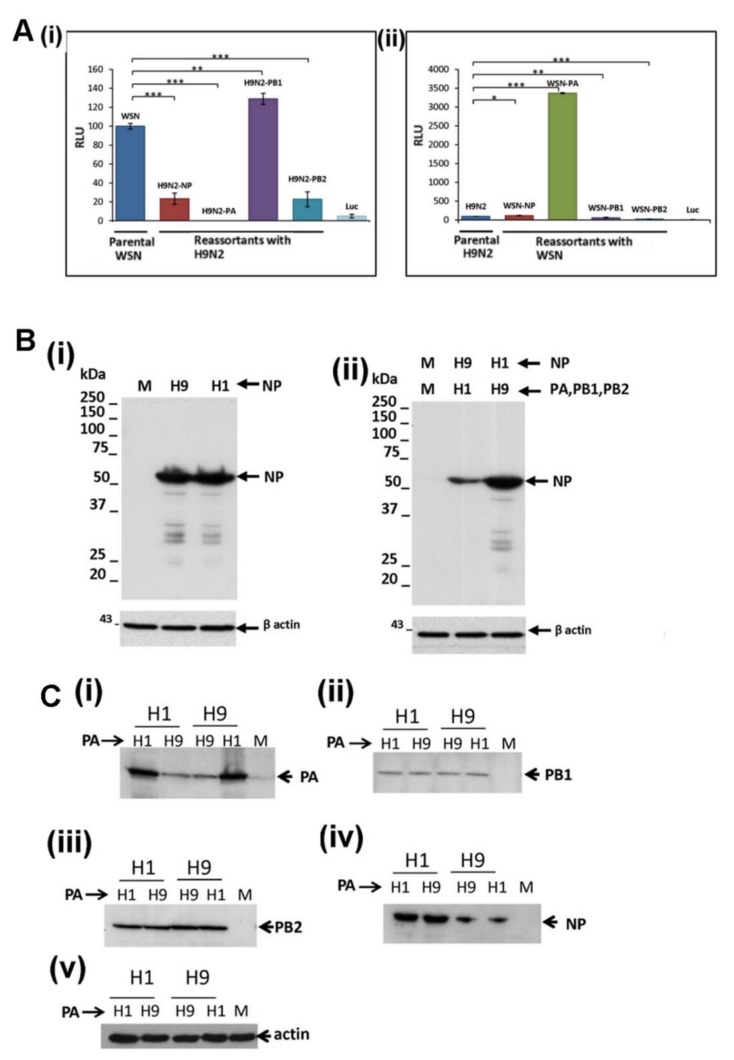
Activity of hybrid polymerase complexes formed between the H1N1/WSN and H9N2 viruses. (**A**) Cells were co-transfected with the pCAGGS/PA, pCAGGS/PB1, pCAGGS/PB2, and pCAGGS/NP of the (i) H1N1/WSN and (ii) H9N2 viruses and then replaced in turn with the corresponding pCAGGS/PA, pCAGGS/PB1, pCAGGS/PB2, pCAGGS/NP of (i) the H9N2 and (ii) the H1N1/WSN viruses. The activity was measured by recording the luciferase at 4 days post-transfection. Each assay was performed in triplicate and representative data from one experiment is shown. The data was analyzed using the student T-test. Pairwise analysis values: * *p* < 0.05; ** *p* < 0.005; *** *p* < 0.001. (**B**) Cells were (i) singly transfected with the pCAGGS/NP of the H1N1/WSN (H1) and H9N2 (H9) virus or (ii) co-transfected with the H1N1/WSN (H1) virus pCAGGS/PA, pCAGGS/PB1, pCAGGS/PB2, and the H9N2 (H9) virus pCAGGS/NP or co-transfected with H9N2 (H9) virus pCAGGS/PA, pCAGGS/PB1, pCAGGS/PB2, and the H1N1/WSN pCAGGS/NP. At 24 hrs post-transfection the cells were immunoblotted with anti-NP. The location of the NP is indicated (black arrow) and actin is a loading control. Cells transfected with pCAGGS are also shown (M). **(C)** Cells were co-transfected with the pCAGGS/PB1, pCAGGS/PB2, pCAGGS/NP of the (H1) H1N1/WSN and (H9) H9N2 viruses and the pCAGGS/PA of the H1N1/WSN virus (H1) or H9N2 virus (H9). At 24 h post-transfection the cells were immunoblotted with (i) anti-PA, (ii) anti-PB1, (iii) anti-PB2 and (iv) anti-NP. Protein bands corresponding in size to the full-length (black arrow) polymerase proteins are indicated, and (v) actin is a loading control.

It was unclear if the reduced expression of the H9N2 virus NP and PA protein could be mitigated by co-expression of the H9N2 and H1N1/WSN virus polymerase proteins, and we examined the properties of the hybrid H1N1/WSN and H9N2 virus recombinant polymerase complexes. While the H1N1/WSN virus polymerase complex demonstrated high levels of polymerase activity, the hybrid H1N1/WSN virus polymerase complex containing the H9N2 virus NP exhibited an approximate 80% reduction in the polymerase activity (Figure 6A(i)). In contrast, the H9N2 virus polymerase complex demonstrated low levels of polymerase activity, and the hybrid H9N2 virus polymerase complex containing the H1N1/WSN virus NP exhibited a 20% increase in the polymerase activity (Figure 6A(ii)). Immunoblotting with anti-NP indicated low NP levels in cells expressing the hybrid H1N1/WSN virus polymerase complex with the H9N2 virus NP (Figure 6B(i),(ii)). This indicated that co-expression of the H1N1/WSN polymerase proteins did not mitigate the low levels of H9N2 virus NP expression.

The hybrid H1N1/WSN virus polymerase complex containing the H9N2 PA protein exhibited less than 0.1% of the polymerase activity of the parental H1N1/WSN virus polymerase complex (Figure 6A(i)), and this was similar to the polymerase activity of the parental recombinant H9N2 virus polymerase complex. In contrast, the hybrid H9N2 virus polymerase complex containing the H1N1/WSN virus PA protein exhibited an approximately 32-fold increase in polymerase activity (Figure 6A(ii)), and its polymerase activity was comparable to the parental H1N1/WSN virus polymerase complex. We examined the effect of exchanging the PA protein on the expression of the other polymerase proteins within the hybrid H1N1/WSN and H9N2 virus polymerase complexes by immunoblotting with anti-PA, anti-PB2, anti-PB1 and anti-NP (Figure 6C). This indicated that the replacement of the PA protein in each hybrid virus polymerase complex had no effect on the expression levels of the NP and other virus polymerase proteins. This also indicated that the reduced H9N2 virus PA protein expression level was not reversed by its co-expression with the H1N1/WSN virus polymerase proteins. Collectively these data demonstrated that the reduced NP and PA protein levels observed in the H9N2 virus-infected cells occurred independently of virus infection and was an intrinsic property of these proteins. This intrinsic property of the H9N2 PA protein expression is unlikely to be solely attributable to its avian origin. The pH1N1 and H9N2 virus PA protein contained known avian signatures motifs (Supplementary Figure S4), which was consistent with the avian origin of the PA gene of both these viruses. However, the expression levels of the pH1N1 virus PA protein were higher than that of the H9N2 virus PA protein, being comparable to the PA protein expression levels in H1N1/WSN virus-infected cells.

Comparable H1N1 and H9N2 virus PB1 and PB2 protein levels were detected in both infected cells (Figure 1) and in the minireplicon system described above (Figure 5B(i),(ii); Supplementary Figure S3C,D). The hybrid H1N1/WSN polymerase complex containing the corresponding PB2 protein of the H9N2 virus exhibited an 80% reduction in the polymerase activity but was not associated with reduced protein levels (Figure 6A). This was consistent with PB2 protein-specific sequence motifs that influenced the replication of the avian viruses in human cells e.g., [23,41].

### 3.5. Efficient Expression of the PA Protein Facilitates Nuclear Export of the RNP Complex

Newly synthesized virus protein (e.g., the NP, PA protein) in a single cycle of infection is primarily due to the transcriptional activity of the RNP complex from the incoming challenge virus [42]. We hypothesized that the reduced H9N2 virus PA protein levels could lead to the formation of a proportion of incompletely formed RNP complexes in the nucleus of infected cells which could exhibit impaired nuclear export. We have previously demonstrated that the H1N1/WSN NP and the polymerase proteins are efficiently expressed in MDCK cells. The NP was detected at between 2 and 4 hpi, while the polymerase proteins were detected at between 4 and 6 hpi [34]. The appearance of these proteins was concomitant with efficient nuclear export of the H1N1/WSN virus NP at between 4 and 6 hpi [33]. We, used this well-characterized virus-cell system to examine the effect of artificially reducing PA protein expression on the RNP nuclear export in infected cells using siRNA. We have also observed impaired nuclear export of the RNP complexes in H9N2 virus-infected MDCK cells [33], indicating that the MDCK cell model was suitable for examining the effect of reduced PA protein expression on RNP nuclear export. In addition, we noted that MDCK cells exhibited higher siRNA transfection efficiencies when compared with the A549 cells. Three siRNA molecules were used in this analysis; siGFP which targeted the green fluorescent protein (GFP) mRNA (a negative control), and siNP and siPA that targeted the H1N1/WSN NP and PA mRNA respectively [37,43]. Using the siGlo probe we estimated that approximately 85% transfection efficiencies were routinely attained in MDCK cells, and that siPA treatment routinely lead to an approximate 90% reduction in PA protein expression levels [34].

**Figure 7:**
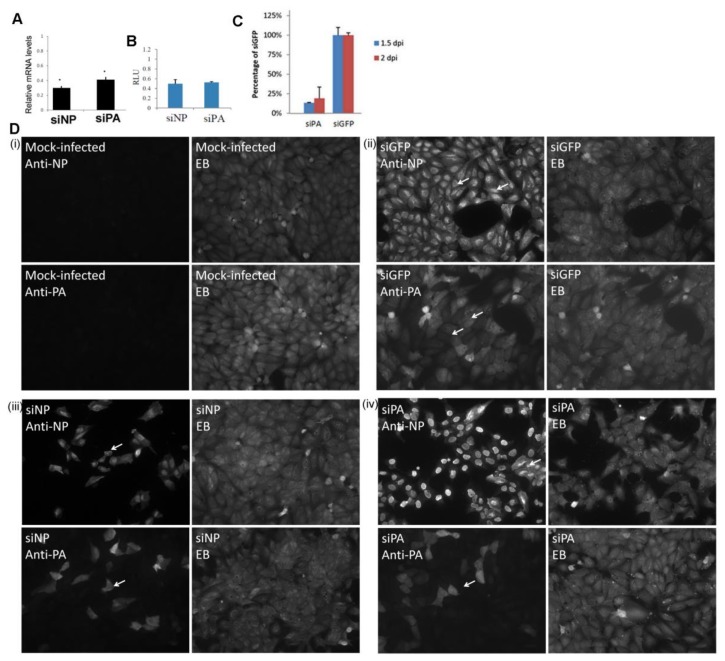
Impaired nuclear export of the RNP occurs after reducing PA protein levels. (**A**) MDCK cells were treated with siNP or siPA and infected with H1N1/WSN using a multiplicity of infection (MOI) of 5. After 12 h post-infection (hpi) the levels of the NP and PA mRNA in the siNP and siPA levels were determined by qPCR, and the data is presented as the level of NP and PA mRNA compared with that in siGFP-treated cells. * *p* > 0.05. (**B**) HEKT293 cells were treated with siNP, siPA or siGFP and transfected with the H1N1/WSN virus pCAGGS/PA, pCAGGS/PB1, pCAGGS/PB2, pCAGGS/NP and the pPol1/luc. The activity of the polymerase complex was assayed after 4 days, and each assay was performed in triplicate and is expressed as luminescence units (RLU). The RLU values indicated are relative to that of siGFP-treated cells **(C)** MDCK cells were treated with siPA or siGFP and infected with H1N1/WSN using an MOI of 0.1. At 1.5 and 2 days-post-infection (dpi) the infectivity in the supernatant was recovered and assayed by plaque assay. Each assay was performed in triplicate. (**D**) Cells were either (i) mock-infected or infected with H1N1/WSN using a MOI of 5 and treated with (ii) siGFP, (iii) siNP or (iv) siPA. At 16 hpi the cells were stained with anti-NP or anti-PA, and Evans Blues (EB). The cells were imaged using fluorescence microscopy (objective ×20 magnification) and infected cells (white arrows), and nucleus-retained NP (*) are highlighted in (iv).

MDCK cells were treated with siGFP, siNP or siPA and infected with H1N1/WSN and the NP and PA mRNA levels were assessed using qPCR (Figure 7A). An approximate 60 and 70% reduction in the PA and NP mRNA levels were observed in siPA and siNP-treated cells respectively. The siGFP treatment had no significant effect on the polymerase activity in the H1N1/WSN minireplicon assay, but the siNP and siPA treatment led to a 70% and 60% reduction in polymerase activity, respectively (Figure 7B). The antiviral effect of siPA treatment was confirmed on siGFP or siPA-treated cells infected with H1N1/WSN, where reduced virus infectivity was recovered from the siPA-treated cells at 1.5 and 2 days post-infection (Figure 7C).

We used imaging to access the effect of siPA treatment on the nuclear export of the newly formed RNP complexes. MDCK cells were either mock-infected, or treated with siGFP, siNP and siPA, and then infected with H1N1/WSN. The cells were stained using anti-NP and anti-PA, and examined using IF microscopy (Figure 7D). Co-staining with Evan’s Blue was also employed that allowed the detection of both infected and non-infected cells. We failed to detect anti-NP or anti-PA staining in mock-infected cells (Figure 7D(i)), while in siGFP-treated H1N1/WSN virus-infected cells greater than 95% of the cells exhibited anti-NP and anti-PA staining (Figure 7D(ii)). Extensive cytoplasmic anti-NP staining indicated efficient nuclear export of the H1N1/WSN virus RNP complex. In siNP-treated H1N1/WSN virus-infected cells approximately 30% of the cells exhibited anti-NP staining (Figure 7D(iii)), indicating silencing of newly expressed NP. The siNP treatment also led to an approximate 70% reduction in anti-PA protein stained cells, suggesting that the newly expressed NP protein may be required for either PA protein expression or for the stabilization of the newly expressed PA protein. In the siPA-treated cells, approximately 30% of the cells exhibited anti-PA staining indicating silencing of the PA protein expression (Figure 7D(iv)). While greater than 95% of the cells exhibited anti-NP staining in siPA-treated cells, approximately 80% of the cells the anti-NP staining was extensively localized to the nucleus. Therefore, while siPA treatment did not inhibit new NP protein expression in the H1N1/WSN virus-infected cells (Figure 7D(iv)), it did lead to an accumulation of NP in the nucleus. These data provided evidence that efficient expression of the PA protein may be required for efficient nuclear export of RNP complexes. These observations were also consistent with the newly made NP being expressed by the polymerase complex of the input virus, and was consistent with the earlier observations by Bean and Simpson [42].

### 3.6. The H9N2 Virus RNP Complex is Exported from the Nucleus Via a Functional Nuclear Pore Complex

**Figure 8:**
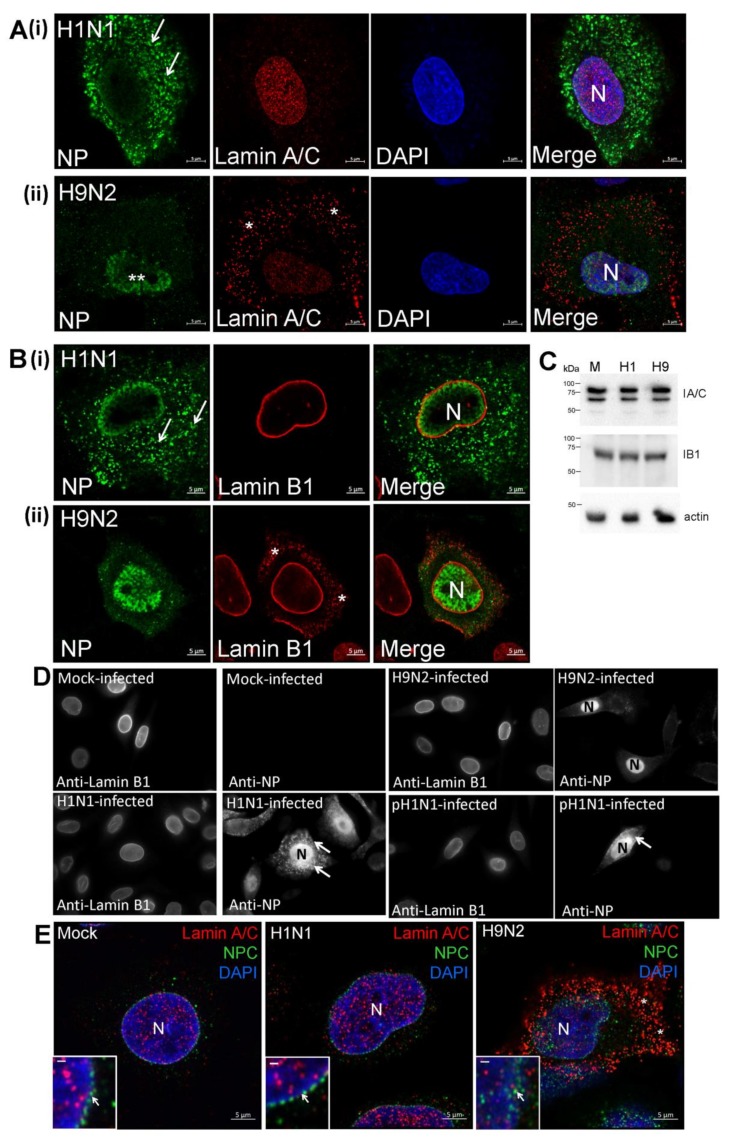

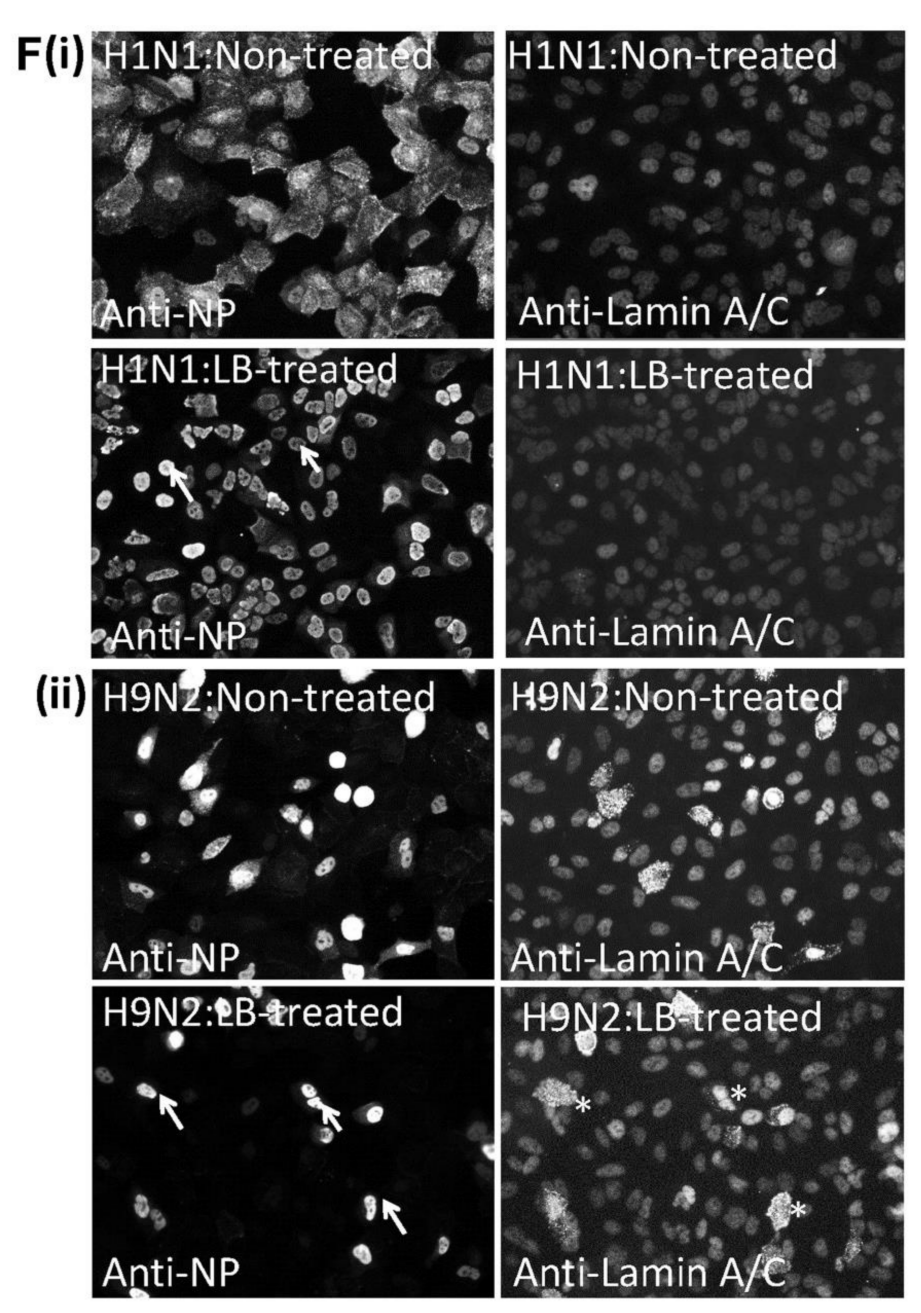
Altered integrity of the nuclear envelope occurs in H9N2 virus-infected A549 cells. A549 cells were infected with (i) H1N1/WSN (H1N1) and (ii) H9N2 influenza viruses using a multiplicity of infection (MOI) of 5. At 20 h post-infection (hpi) the cells were stained using (**A**) anti-NP, DAPI and anti-lamin A/C or (**B**) anti-NP and anti-lamin B1, and examined using confocal microscopy. The location of the nucleus (N), H9N2 virus NP retained in the nucleus (**) and cytoplasmic anti-lamin A/C and B1 staining (*) are highlighted. (**C**) Cell lysates were prepared from (M) mock-infected, (H1) H1N1/WSN virus-infected and (H9) H9N2 virus-infected A549 cells and immunoblotted using anti-lamin A/C (lA/C), anti-lamin B1 (lB1) and anti-actin (loading control). Protein species corresponding in size to lamin A/C, lamin B1 and actin are indicated. (**D**) Cells were mock-infected or infected with the H1N1, H9N2, and pH1N1 viruses, and at 12 hpi the cells were co-stained using anti-NP and anti-lamin B1, and imaged using fluorescence microscopy (objective x100 magnification). The nucleus (N) and cytoplasmic anti-NP staining (white arrow) are highlighted. (**E**) Mock-infected or cells infected with the H1N1/WSN and H9N2 influenza virus at 20 hrs post-infection (hpi) were stained using anti-NPC (green), DAPI (blue) and anti-lamin A/C (red), and imaged using confocal microscopy. The nucleus (N) and cytoplasmic anti-lamin A/C staining in H9N2 virus-infected cells (*) are indicated. The insets are higher magnification images showing the demarcation of the nuclear envelope highlighted by the anti-NPC staining (white arrow); bar = 0.5μm. (**F**) Cells were infected with (i) H1N1/WSN or (ii) H9N2 influenza virus using an MOI of 5 and at 2 hpi the cells were either non-treated or treated with leptomycin B (LB-treated). At 18 hpi the cells were co-stained using anti-NP and anti-lamin A/C, and imaged by fluorescence microscopy (objective x20 magnification). The cytoplasmic anti-lamin A/C staining in infected cells (*) and anti-NP stained nuclei (white arrows) are highlighted.

A549 cells were either mock-infected or infected with the H1N1 and H9N2 viruses, and co-stained using anti-NP and anti-lamin A/C (Supplementary Figure S5A) The H1N1/WSN virus-infected cells exhibited a distinct nuclear-like anti-lamin A/C staining pattern similar to that observed in mock-infected cells. In H9N2 virus-infected cells an additional cytoplasmic anti-lamin A/C staining pattern was also apparent. This staining pattern was confirmed using confocal microscopy to examine representative H1N1/WSN and H9N2 virus-infected A549 cells co-stained with anti-NP, DAPI and anti-lamin A/C (Figure 8A). A distinct anti-lamin A/C pattern that coincided with the DAPI staining was noted in cells infected with either virus (Figure 8A(i)), while the additional non-nuclear-like anti-lamin A/C staining in H9N2 virus-infected cells was confirmed (Figure 8A(ii)). The cytoplasmic lamin A/C staining pattern was also not observed in A549 cells infected with either the H4N1 or H7N1 viruses (Supplementary Figure S5B), suggesting that the appearance of the cytoplasmic lamin A/C in H9N2 virus-infected cells was not solely attributable to its avian origin. In a similar analysis, A549 cells were either mock-infected or infected with the H1N1, H9N2 (Supplementary Figure S5C) and pH1N1 (Supplementary Figure S5D) viruses, and then co-stained using anti-NP and anti-lamin B1. The H9N2 virus-infected cells exhibited a cytoplasmic anti-lamin B1 staining pattern that was absent in either H1N1 or pH1N1 virus-infected cells, and analysis by confocal microscopy more clearly showed the different staining patterns in anti-NP and anti-lamin B1 co-stained H1N1 and H9N2 virus-infected A549 cells (Figure 8B). Immunoblotting of cell lysates with anti-lamin A/C and lamin B1 revealed similar levels of full-length lamin A/C and lamin B1 in mock-infected and virus-infected cells (Figure 8C), indicating that there was no change in the total lamin protein levels following virus infection. The absence of smaller lamin protein products in the lysates prepared from the H9N2 virus-infected cells was consistent with the absence of proteolytic degradation of the lamin proteins. The H1N1, H9N2 and pH1N1 virus-infected A549 cells were co-stained with anti-NP and anti-lamin B1 at 12 hpi and examined by IF microscopy (Figure 8D), which was sufficient time for NP expression to be detected in cells infected with each virus. In the H9N2 virus-infected cells the anti-NP staining was restricted to the nucleus, while varying degrees of nuclear export occurred in H1N1 and pH1N1 virus-infected cells. However, in all cases only anti-lamin B1 staining in the nucleus was noted, indicating that the changes in the nuclear envelope in H9N2 virus-infected cells did not occur at the early stages of H9N2 virus infection (e.g., prior to the onset of nuclear export of the RNP complexes).

Previous studies using the established TUNNEL (TUN) staining assay had demonstrated that H9N2 viruses can induce apoptosis in human airway cells [44], and we also examined if the altered lamin distribution in H9N2 virus-infected cells correlated with the onset of apoptosis using TUN-staining and propidium iodide (PI) staining. Mock-infected cells, and H1N1 and H9N2 virus-infected cells were also co-stained using TUN and anti-NP, and examined by IF microscopy (Supplementary Figure S6A,B). Although sporadic TUN-stained cells were observed in all three experimental conditions, increased TUN staining in H9N2 virus-infected cells was not observed. This suggested that apoptosis may not account for the change in lamin distribution. In fixed cells that were permeabilized with triton X-100 and stained with PI, both mock-infected and H1N1 virus-infected cells exhibited PI-stained nuclei (Supplementary Figure S6C), while increased PI staining in the nucleus of H9N2 virus-infected cells was noted. The absence of increased TUN-staining in H9N2 virus-infected cells suggested that the increased PI staining in the nucleus was unlikely due to strand breaks in nuclear DNA arising from DNA damage [45], but it may indicate changes in chromatin structure that lead to increased DNA exposure and staining. While PI staining of live mock-infected and H1N1 virus-infected cells was not detected, PI-staining in the live H9N2 virus-infected cells indicated increased membrane permeability in these cells (Supplementary Figure S6D), which suggested H9N2 virus-induced cell cytotoxicity. Although the appearance of the lamins in the cytoplasm did not appear to involve an apoptotic mechanism, this phenomenon correlated with the onset of virus-induced cytotoxicity.

We examined if the nuclear export of the H9N2 virus RNP complex involved a functional nuclear pore complex (NPC). Mock-infected cells, and H1N1 and H9N2 virus-infected A549 cells were co-stained with anti-lamin A/C and anti-NPC, and the cells imaged by IF microscopy (Supplementary Figure S7A,B). Although cytoplasmic anti-lamin A/C staining was observed in H9N2 virus-infected cells, the anti-NPC staining in the nucleus was consistent with an intact nuclear envelope. Mock-infected cells, and H1N1 and H9N2 virus-infected cells were co-stained using anti-lamin A/C, anti-NPC and DAPI and examined in greater detail using confocal microscopy (Figure 8E). The anti-NPC staining pattern in both mock-infected and H1N1 virus-infected cells suggested an intact nuclear envelope, and while the NPC staining in H9N2 virus-infected A549 cells was less well-defined, the staining pattern was also consistent with an intact nuclear envelope. In addition, immunoblotting of nuclei isolated from H9N2 virus-infected A549 cells with anti-NPC indicated no reduction in the levels of the NPC when compared with mock-infected and H1N1 virus-infected cells (Supplementary Figure S7C).

Leptomycin B (LB) inhibits the CRM1 protein [46] and prevents the nuclear export of RNP complexes via the NPC. A549 cells were infected with the H1N1 and H9N2 viruses, and at 3 hpi the cells were either non-treated or LB-treated. At 18 hpi the cells were co-stained using anti-NP and anti-lamin A/C and imaged by IF microscopy (Figure 8F). In the non-treated H1N1 virus-infected cells efficient nuclear export of the NP protein in H1N1 virus-infected cells was noted (Figure 8F(i). In the H9N2 virus-infected cell monolayer the cytoplasmic anti-lamin B1 staining was only detected in infected cells (Figure 8F(ii)), indicating that the change in lamin distribution was directly related to virus infection and did not involve external trans-acting factors such as e.g., secreted cytokines. As observed previously, between 5 and 10% of the H9N2 virus-infected cells exhibited NP nuclear export (Figure 8F((ii)). In contrast, in the LB-treated cells infected with both the H1N1 and H9N2 viruses the NP staining was entirely confined to the nucleus and suggested inhibition of RNP nuclear export. This indicated that the low level of nuclear export of the H9N2 virus RNP complex involved a functional NPC despite the changes in the nuclear envelope. These data also suggested that in H9N2 virus-infected cells the lamin A/C protein translocation into the cytoplasm did not involve active transport through the NPC. Furthermore, cytoplasmic lamin A/C was not detected in LB-treated H1N1-virus infected cells suggesting that the increased NP levels in the nucleus of H9N2 virus-infected cells were not in itself the direct cause for the transfer of lamin A/C into the cytoplasm.

### 3.7. Efficient Export of the H9N2 Virus RNP Complex in Avian Cells Mitigates Virus-Induced Cytotoxicity in CEF Cells

**Figure 9:**
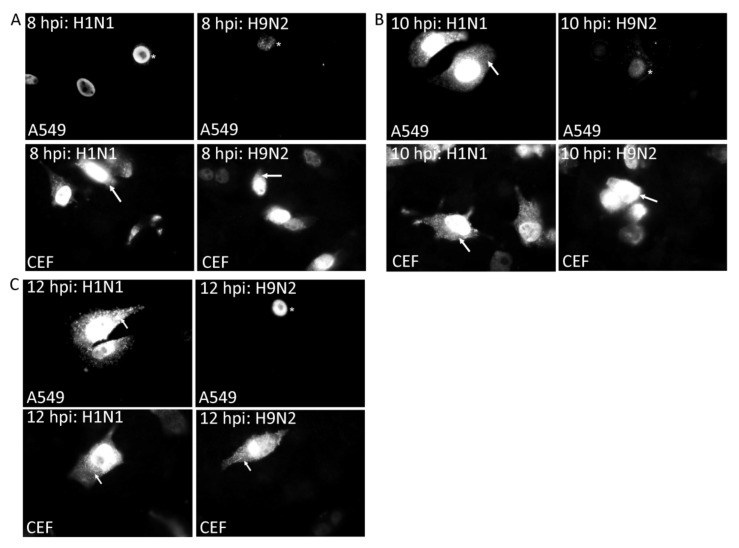
Nuclear export of the RNP complex during the early stages of virus infection. A549 and CEF cells were infected with H1N1 and H9N2 viruses and at (**A**) 8, (**B**) 10 and (**C**) 12 h post-infection the cells were stained using anti-NP and imaged by fluorescence microscopy using identical camera settings (objective x100 magnification). The cytoplasmic (white arrow) and nuclear (*) anti-NP staining are highlighted.

While the H1N1/WSN NP can be detected at between 2 and 4 hpi in mammalian cells, the expression of the H9N2 virus NP occurs much later. It can only be detected using immunoblotting by 10 hpi, with increased NP levels being detected up to 20 hpi (Myaing and Sugrue, unpublished data). The temporal expression and nuclear export of the NP protein was therefore examined in H1N1 and H9N2 virus-infected A549 and CEF cells at between 8 and 12 hpi. The anti-NP stained cells were imaged using IF microscopy to distinguish nuclear and non-nuclear NP staining (Figure 9). While the delayed nuclear export and lower expression levels of the H9N2 virus NP occurred in A549 cells, comparable levels of NP protein expression and efficient NP nuclear export were noted for both viruses in CEF cells from 8 hpi. These data highlighted the impaired nuclear export of the H9N2 virus RNP complex relatively early in infection in A549 cells, and that concomitant NP expression and efficient nuclear export of the RNP complex occurs relatively early in CEF cells infected with either virus.

**Figure 10:**
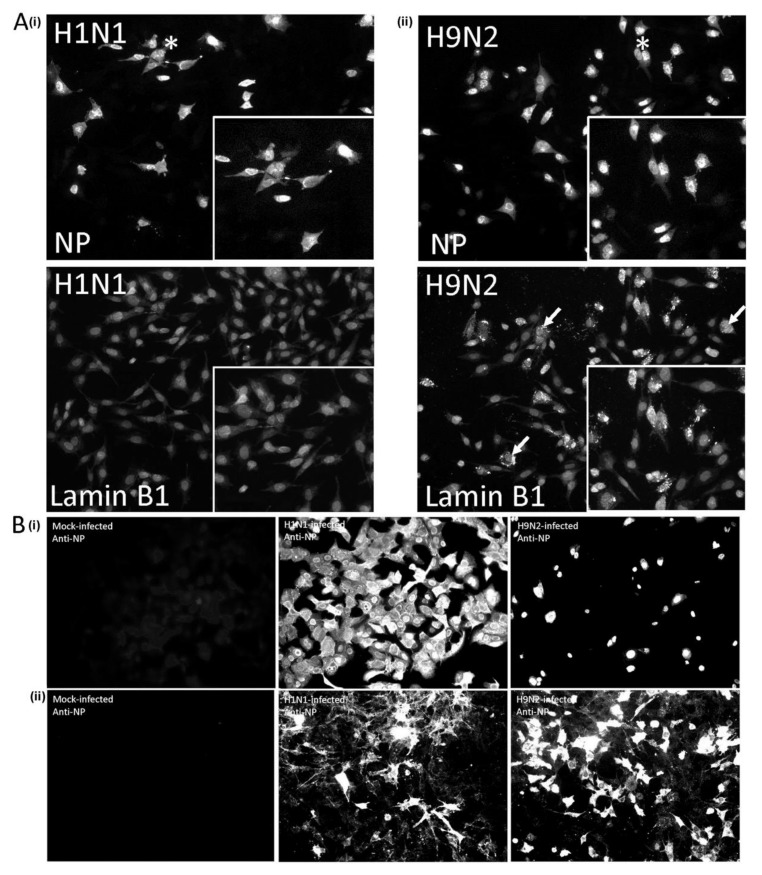
Impaired H9N2 virus transmission occurs in virus-infected A549 cells but not CEF cells. (**A**) CEF cells were infected with (i) H1N1/WSN and (ii) H9N2 viruses using a multiplicity of infection (MOI) of 0.1 and at 18 hr post-infection (hpi) the cells were co-stained with anti-NP and anti-lamin B1, and imaged using fluorescence microscopy (objective ×20 magnification). The altered anti-lamin B1 straining is highlighted (white arrow). Insets are enlarged areas highlighted by * in the main plate (**B**) (i) A549 cells and (ii) CEF cells were mock-infected or infected with the H1N1/WSN and H9N2 viruses using an MOI of 0.05 in DMEM containing BSA and 1μg/mL TPKC trypsin. At 36 hpi the cells were stained using anti-NP and imaged using fluorescence microscopy (objective x20 magnification).

CEF cells were infected with H1N1 and H9N2 viruses, and stained using anti-NP and anti-lamin B1. Examination of the stained cells by IF microscopy showed that in both cases there was efficient export of the virus NP (Figure 10A (i) and (ii)) consistent with the data presented above (Figure 9). However, in H9N2 virus-infected CEF cells the cytoplasmic anti-lamin B1 staining was also noted (Figure 10A(ii)). This suggested that the virus-induced cellular changes that led to the cell cytotoxicity were independent of host species from which the cells were derived i.e., it occurred in human and avian origin cells infected with the H9N2 virus.

We also examined H1N1 and H9N2 virus infection in a multiple-cycle virus infection model in A549 and CEF cells. Cells were infected with the H1N1 and H9N2 virus using an MOI of 0.05 and at 36 hpi the cells were co-stained using anti-NP and anti-lamin B1, and imaged by IF microscopy (Figure 10B). Approximately 60% of the cells in the H1N1 virus-infected A549 cell monolayer showed anti-NP staining indicating efficient spread across the cell monolayer (Figure 10B(i)). In contrast, approximately 10% of the cells the H9N2 virus-infected A549 cell monolayer displayed intense anti-NP staining, which was consistent with the impaired spread of H9N2 virus infection. While we were able to detect low levels of the cell-free H1N1 virus in the tissue culture media (3.9 × 10^3^ pfu/mL), we failed to detect the presence of cell-free H9N2 virus in the tissue culture of infected. Examination of anti-NP stained CEF cells infected with either virus exhibited widespread staining across the cell monolayer, consistent with the efficient spread of infection (Figure 10B(ii)), and in both cases cell-free virus could be detected in the tissue culture media (H1N1: 5.2 × 10^5^ pfu/mL; H9N2: 4.6 × 10^4^ pfu/mL). These data are consistent with our previous observations [33], and suggested that the efficient nuclear export of the RNP complex in H9N2 virus-infected CEF cells was able to mitigate the virus-induced cytotoxic effects.

## 4. Discussion

Although there was a delay in the onset of H9N2 virus gene expression, the virus was able to infect the A549 cells used in this study. In contrast to the productive infection by the H1N1 virus in these cell types, the H9N2 produced an abortive infection in these cells. The biological properties of the H9N2 virus polymerase complex appeared to be a major factor that restricted the propagation of the H9N2 virus in these cells. The reconstitution of the H9N2 virus polymerase complex in the absence of infection leads to reduced NP protein levels, suggesting that the formation of the H9N2 virus polymerase complex may initiate cellular changes that in turn lead to reduced NP protein levels. Expression of TRIM22 induced by interferon-α has been previously reported to target the NP for degradation [47], and reports using recombinant NP expression have suggested that the NP can be targeted by ubiquitination [48]. The H9N2 virus is capable of initiating a robust antiviral response in A549 cells, but neither increased interferon-α or TRIM22 gene expression in H9N2 virus-infected A549 cells was detected [33]. Similarly, H9N2 virus NP protein species with an increased molecular mass that would be expected following ubiquitination and these were not detected. Reconstitution of an active replication complex may be sufficient to initiate antiviral responses e.g., interferon signaling via the RNA sensor RIG I [49]. However, this generalized antivirus response would not be expected to specifically target the NP protein expression. It ispossible that the activation of another unidentified and more specific cellular pathway may lead to the down-regulated expression of the H9N2 virus NP protein. However, the most noticeable effect of the reduced NP levels was a reduction in the activity of the H9N2 virus polymerase complex.

The reduced expression of the H9N2 virus PA protein in non-avian cells was observed in the absence of infection and therefore suggested that this was an intrinsic property of the H9N2 PA protein. A list of sequence variants that are associated with avian adaptation has been proposed [50,51,52], and the same avian sequence motifs are present in the pH1N1/471 and H9N2 PA proteins.

Since similar PA protein levels were detected in pH1N1/471 and H1N1/WSN virus-infected cells, it is unlikely that these known avian-specific sequences are the cause of the reduced H9N2 virus PA protein levels. Reduced H9N2 virus PA protein expression may be mediated by other sequence motifs within the H9N2 virus PA mRNA that either directly or indirectly impact on the PA protein levels. For example, the rate of protein translation can determine protein levels in cells, and this is determined by several factors [53], which include the adaptation to tRNA pools, amino acid composition of the expressed protein and the mRNA folding energy [54]. Translation of influenza virus mRNA involves the recruitment of essential host factors [55], and interactions between the PB2 protein and eIF4G have been demonstrated [56,57,58]. It is, therefore, possible that differences in the recruitment of one or more specific host cell factors to the H9N2 virus PA mRNA may account for the reduced PA protein levels. Although the factors that lead to reduced NP and PA protein expression in A549 cells have not be defined, the process occurs post-transcriptionally and is associated with reduced activity of the H9N2 virus polymerase complex.

An additional factor that restricts the propagation of the H9N2 virus in A549 cells is the reduced efficiency of the nuclear export of the RNP complex. It is unlikely that the accumulation of the H9N2 virus NP in the nucleus is directly related to the reduced NP expression levels since efficient nuclear export of the pH1N1 NP occurred despite the reduced pH1N1 NP expression levels. The NEP plays an important role in the nuclear export of newly formed RNP complexes [19], and avian adaptation of the NEP is associated with three amino acid sequence signatures [52]. The H9N2, H4N1 and H7N1 viruses contain all three avian signature sequences, while the pH1N1 contains two of these avian signature sequences. Since the RNP complex appears to be exported from the nucleus of cells infected with these viruses it is unlikely that these known avian signature sequences are entirely responsible for the impaired nuclear export of the H9N2 virus RNP complex. The M1 protein is a major structural component of mature influenza virus particles, but it also forms part of the RNP complex during nuclear export. The M1 protein tethers the NEP to the NP [19], and signature sequences have been identified in the NEP binding domain of the M1 protein that are associated with human and avian adaptation [52]. Interestingly, the pH1N1 and H9N2 viruses both contain M1 protein sequence motifs that are associated with avian origin, suggesting that the avian origin of the H9N2 virus M1 protein is unlikely to be the direct cause of the reduced nuclear export of the RNP complex in human cells. However, artificially reducing the PA protein levels in H1N1/WSN virus-infected MDCK cells also led to impaired nuclear export of RNP complexes. Although we have not examined the effect of using siRNA to reduce the PA protein expression in A549 and CEF cells, the anaysis in MDCK cells provided evidence that the reduced H9N2 virus PA protein expression may also indirectly contribute to the impaired nuclear export RNP complex in A549 cells. The PA protein is required for nuclear import of the PB1 protein [59], and while a nuclear localization sequence within the PA protein has been identified [60], nuclear export signals within the PA protein have not yet been identified. The stoichiometry of the PB1, PB2 and PA proteins in the functional polymerase complex is equimolar [11,16], and we can hypothesize that an RNP complex destined for nuclear export would require the presence of the polymerase proteins in the correct stoichiometry. The reduced PA protein levels may disrupt this stoichiometry and lead to the retention of incorrectly formed RNP complexes in the nucleus. Although this needs to be examined further, in this scenario we speculate that the formation of a polymerase complex with the correct stoichiometry may be a prerequisite for efficient nuclear export of the RNP complex rather than the PA protein playing a specific role in the nuclear export process. Interestingly and consistent with this interpretation, we have also observed impaired nuclear export of RNP complexes in other influenza viruses following the silencing of either the PA, PB1 or PB2 protein expression (Yeo, Tan and Sugrue, unpublished observations). However, in this scenario it is not clear what would be the minmal level of PA protein expression required for efficient nuclear export of the RNP complex, or if it would be the same in cells of human and avian origin.

The impaired nuclear export of the RNP complex in H9N2 virus-infected cells was compounded by virus-induced cytotoxicity, but this did not appear to involve apoptosis. Although nuclear export of the H9N2 virus RNP complex occurred via a functional NPC, the redistribution of the lamin proteins was consistent with a structural modification in the nuclear envelope during H9N2 virus infection. The presence of lamin proteins in the cytoplasm has been observed following degradation of lamin proteins by caspase activity [61], but we found no evidence that lamin degradation in H9N2 virus-infected cells, or that the presence of the cytoplasmic lamin distribution could be prevented by using caspase inhibitors (Sugrue, unpublished observations). The appearance of lamin B1 in the cytoplasm during nuclear autophagy has been reported [62], but the cytoplasmic lamin A/C and B1 proteins in H9N2 virus-infected cells did not co-stain with autophagosome makers such as LC3B (Kumar and Sugrue, unpublished observations). Changes in the distribution of the lamin proteins can also occur during mitosis [63], but under our experimental conditions, we failed to detect significant levels of cells undergoing mitotic cell division during either the H1N1 or H9N2 virus infection. However, changes in the distribution of lamin proteins can also be mediated by other cellular processes that are not directly related to cell division, including virus infection (reviewed in [64]). For example, the HIV1 vpr protein has been previously shown to modify the nuclear lamina network, eventually leading to the rupture of the nuclear envelope [65]. This facilitates the entry of pre-integration complexes into the cell nucleus prior to the integration of the viral cDNA genome into the host cell DNA. Although nuclear rupture can allow transfer of nuclei resident proteins into the cytoplasm, sealing these membrane lesions with existing cellular membranes can repair the damage and ensure a degree of cell viability [64,66,67]. The cytoplasmic lamin staining pattern occurred in leptomycin B-treated H9N2 virus-infected cells suggesting that this process does not involve the crm1 export pathway. It is currently unclear if one or more H9N2 virus-specific proteins can cause structural changes in the nuclear envelope or if this occurs indirectly via virus-induced signaling networks. However, the nuclear accumulation of the H1N1 virus RNP complex following leptomycin B treatment did not lead to the appearance of the lamin proteins in the cytoplasm, indicating that the accumulation of the H9N2 virus NP in the nucleus may not in itself be sufficient to induce changes in the nuclear envelope integrity. Changes in the nuclear envelope do not occur during the early stages of H9N2 virus infection suggesting that these changes are mediated by later events in the virus replication cycle e.g., as the RNP complex interacts with the NPC just prior to its nuclear export. The ordered packaging of the RNPs into progeny involves a modified endoplasmic reticulum (ER) [68,69], and the maintenance of the spatial context between the nuclear envelope and would be expected to facilitate this process. Disruption of the nuclear envelope integrity may therefore also impact the structural integrity of the ER and could impair the packaging of the RNP complexes.

It is interesting to note that similar changes in the nuclear envelope occurred in both A549 and CEF cells infected with the H9N2 virus. This suggests a common mechanism, possibly involving host cell factors, that is conserved in mammalian and avian species infected with the H9N2 virus. However, while delayed export of the RNP complex was noted in A549 cells, earlier and efficient nuclear export of the H9N2 virus RNP complex was noted in CEF cells. This indicates that virus-induced cytotoxicity may not in itself be sufficient to restrict H9N2 virus transmission in A549 cells, but that this arises from a combination of both delayed nuclear export of the RNP complexes and virus-induced cell cytotoxicity. In this paradigm, the effects of the virus-induced cytotoxicity in restricting virus transmission can be mitigated in CEF cells by significant levels of nuclear export of the RNP complexes prior to the onset of advanced cell cytotoxicity.

Our study provides evidence that the reduced expression of the NP and PA proteins in mammalian cells are intrinsic properties of these proteins, and that these properties may represent an additional obstacle during species adaptation of the H9N2 virus in the natural environment. The first and most apparent effect of reduced expression of the NP and PA proteins was the reduced activity of the H9N2 virus polymerase complex. However, our data also suggested that impaired nuclear export of the RNP complex may be an additional indirect consequence of reduced PA protein expression. The H9N2 virus also induces cytotoxicity in infected cells, and these changes are associated with alteration in the structure of the nuclear envelope. The combined effects of the delay in RNP export and the virus-induced cell cytotoxicity appear to over attenuate and restrict H9N2 infection in A549 cells. This clearly demonstrates that host adaptation of the H9N2 virus polymerase complex to a non-avian environment is a multi-factorial process that involves several levels of complexity, including polymerase activity, efficiency of nuclear export of the RNP complex, and virus-induced cytotoxicity. It is currently unclear if the reduced expression of these proteins in human cells is a property shared by other circulating H9N2 avian viruses, but such factors may be an obstacle for the recovery of other virus strains from veterinary specimens using established mammalian cell systems. In a broader context, we can hypothesize that circulating H9N2 viruses that have acquired the PA and NP genes from human-adapted influenza viruses may favor interspecies transmission, while circulating viruses containing the H9N2 virus PA protein or NP would be expected to replicate poorly in a non-avian environment. Sequence analysis of virus genomes is currently used to assess the threat of circulating influenza viruses in the context of zoonotic transmission. Although sequence analysis is an essential tool in surveillance work, our study also highlights the importance of also examining the biological properties of clinical and veterinary influenza virus strains that are circulating in the natural environment. Future studies will focus on defining the molecular mechanisms responsible for reduced H9N2 virus NP and PA protein expression.

## Supplementary Materials

The following are available online at https://www.mdpi.com/2073-4409/9/2/355/s1, Figure S1. Distribution of the RNP and chromatin in the nucleus of H1N1 and H9N2 virus-infected cells; Figure S2. Distribution of the NP in virus-infected A549 cells; Figure S3. Expression of the recombinant polymerase genes of the H1N1/WSN(H1N1), pH1N1/471 (pH1N1) or H9N2(H9N2) viruses; Figure S4. PA protein sequences; Figure S5. Lamin A/C and B1 distribution in influenza virus-infected cells. Figure S6. Increased apoptosis is not observed in H9N2 virus-infected A549 cells; Figure S7 Anti-NPC staining in influenza virus-infected cells; Table S1. List of virus-specific primers used in this study.
