# Supplementary material for: Impaired Nuclear Export of the Ribonucleoprotein Complex and Virus-Induced Cytotoxicity Combine to Restrict Propagation of the A/Duck/Malaysia/02/2001 (H9N2) Virus in Human Airway Cells
Source: Cells. 2020 Feb 3;9(2):355. doi: 10.3390/cells9020355 (PMC7072679; doi:10.3390/cells9020355)
Supplement: Supplementary file 1 [file cells-09-00355-s001.pdf]

Supplementary figures.

SFig. 1

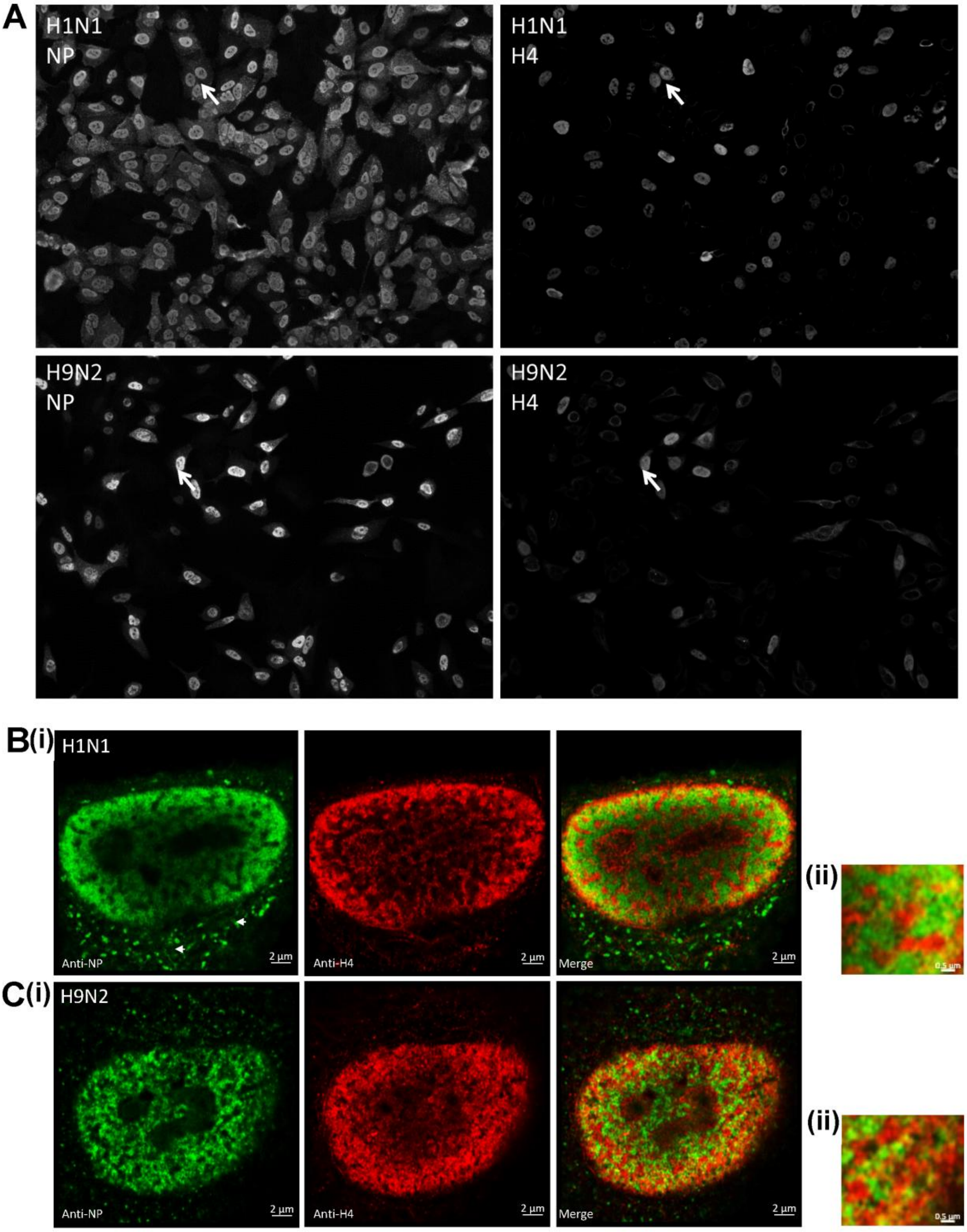

SFig. 2

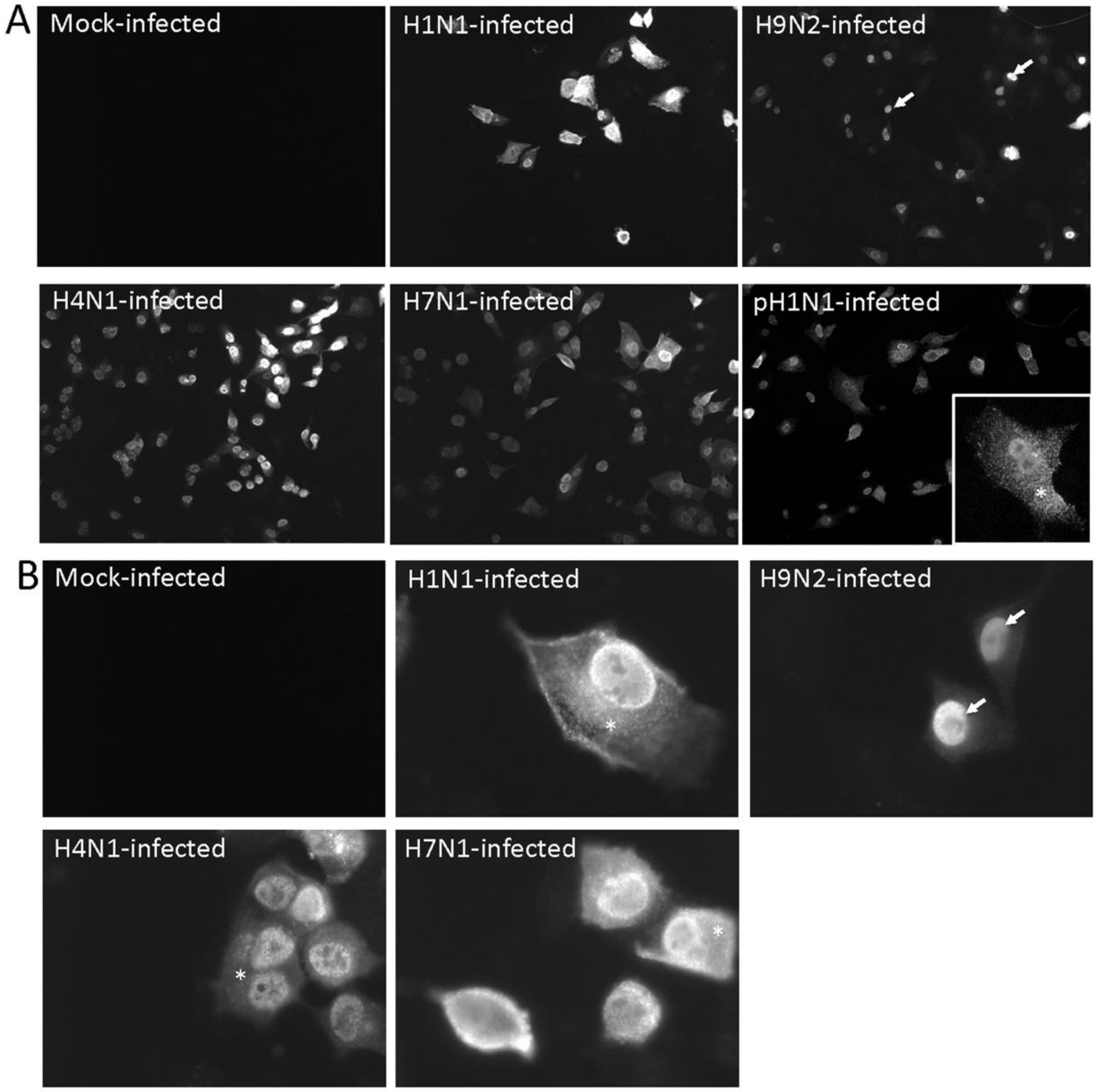
A
Mock-infected
H1N1-infected
H9N2-infected
H4N1-infected
H7N1-infected
pH1N1-infected
B
Mock-infected
H1N1-infected
H9N2-infected
H4N1-infected
H7N1-infected

SFig. 3

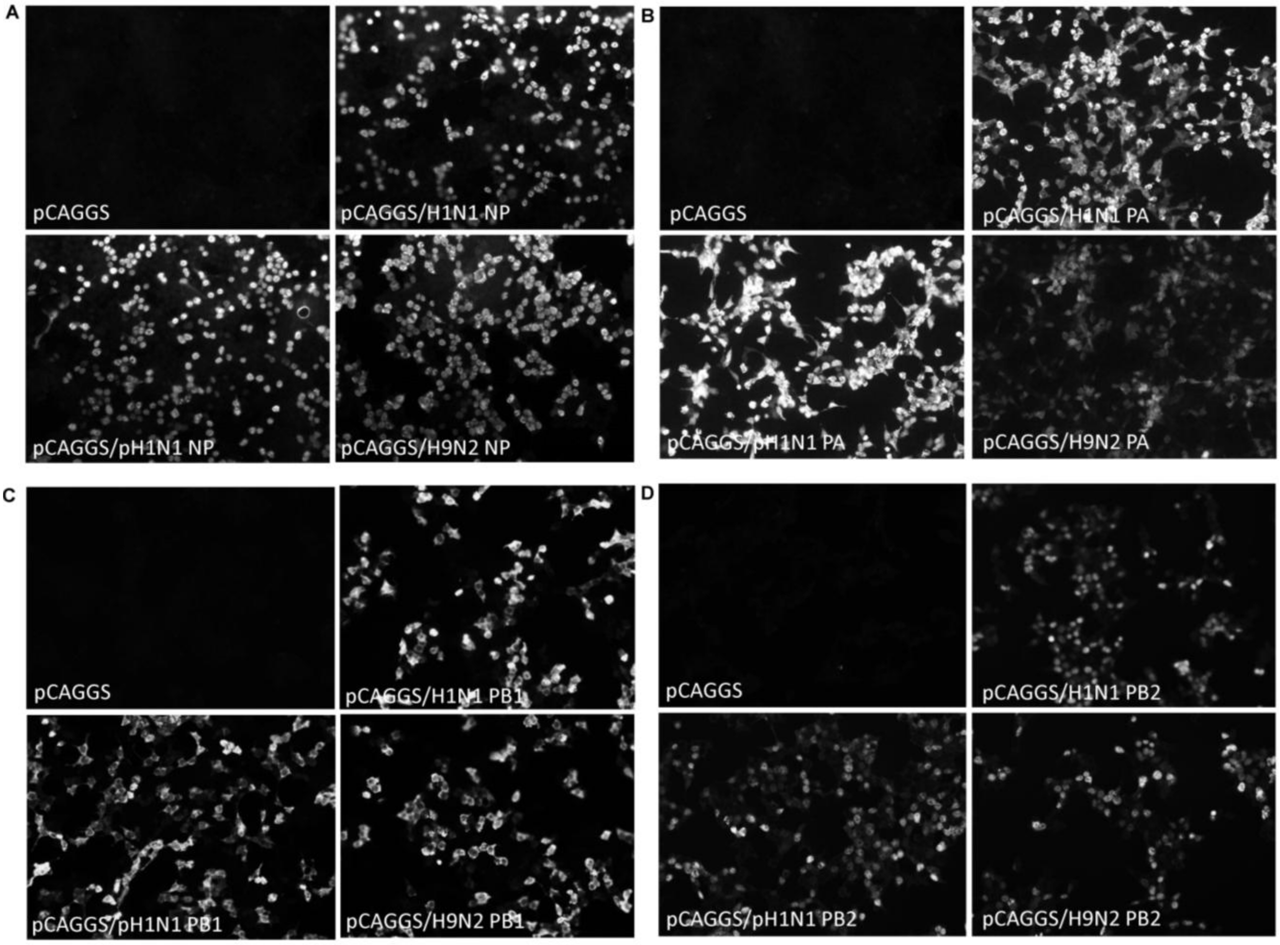
A
pCAGGS
pCAGGS/H1N1 NP
pCAGGS/pH1N1 NP
pCAGGS/H9N2 NP
B
pCAGGS
pCAGGS/H1N1 PA
pCAGGS/pH1N1 PA
pCAGGS/H9N2 PA
C
pCAGGS
pCAGGS/H1N1 PB1
pCAGGS/pH1N1 PB1
pCAGGS/H9N2 PB1
D
pCAGGS
pCAGGS/H1N1 PB2
pCAGGS/pH1N1 PB2
pCAGGS/H9N2 PB2

SFig. 4

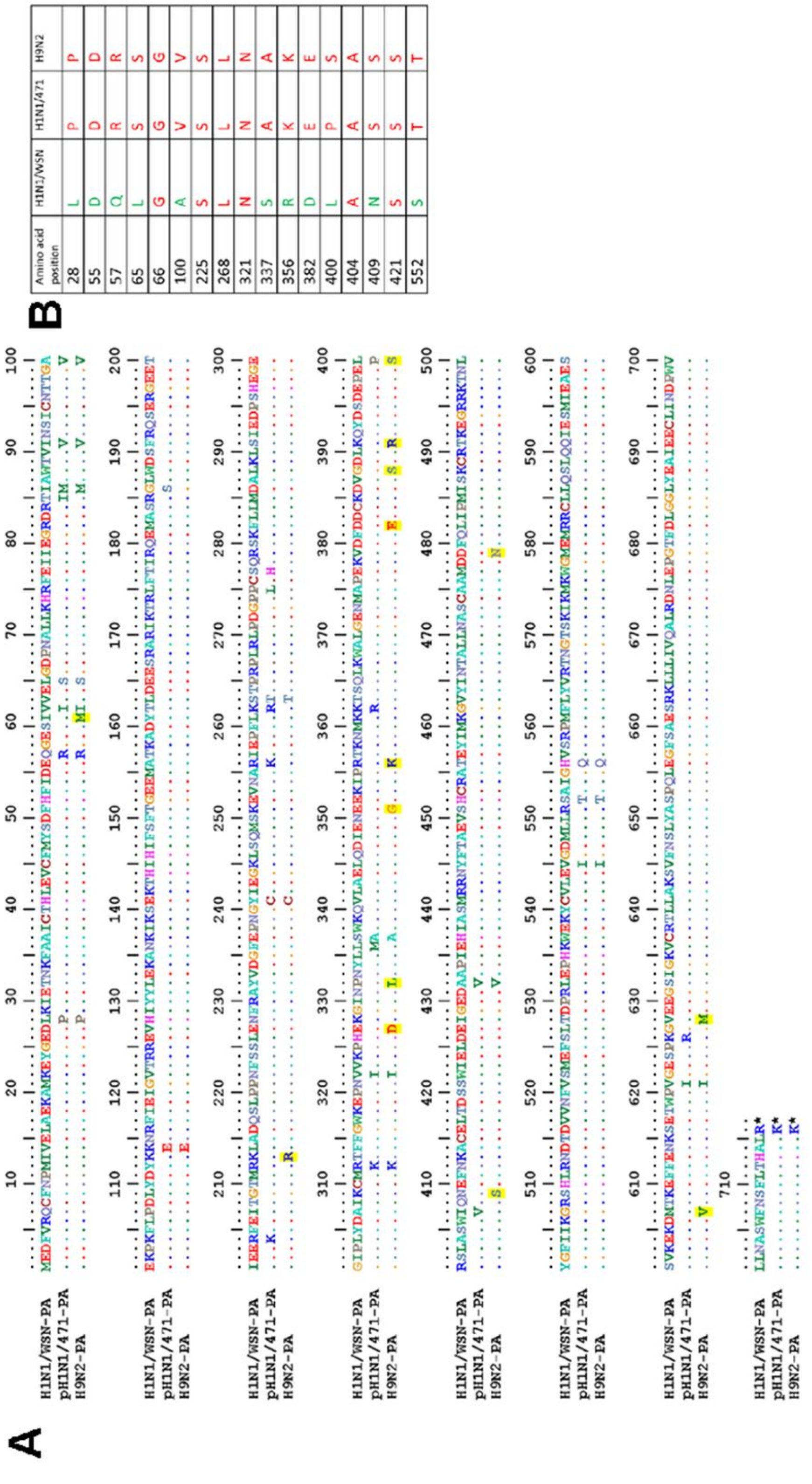

Amino acid position	H1N1/WSN	H1N1/471	H9N2
28	L	P	P
55	D	D	D
57	Q	R	R
65	L	S	S
66	G	G	G
100	A	V	V
225	S	S	S
268	L	L	L
321	N	N	N
337	S	A	A
356	R	K	K
382	D	E	E
400	L	P	S
404	A	A	A
409	N	S	S
421	S	S	S
552	S	T	T

SFig. 5

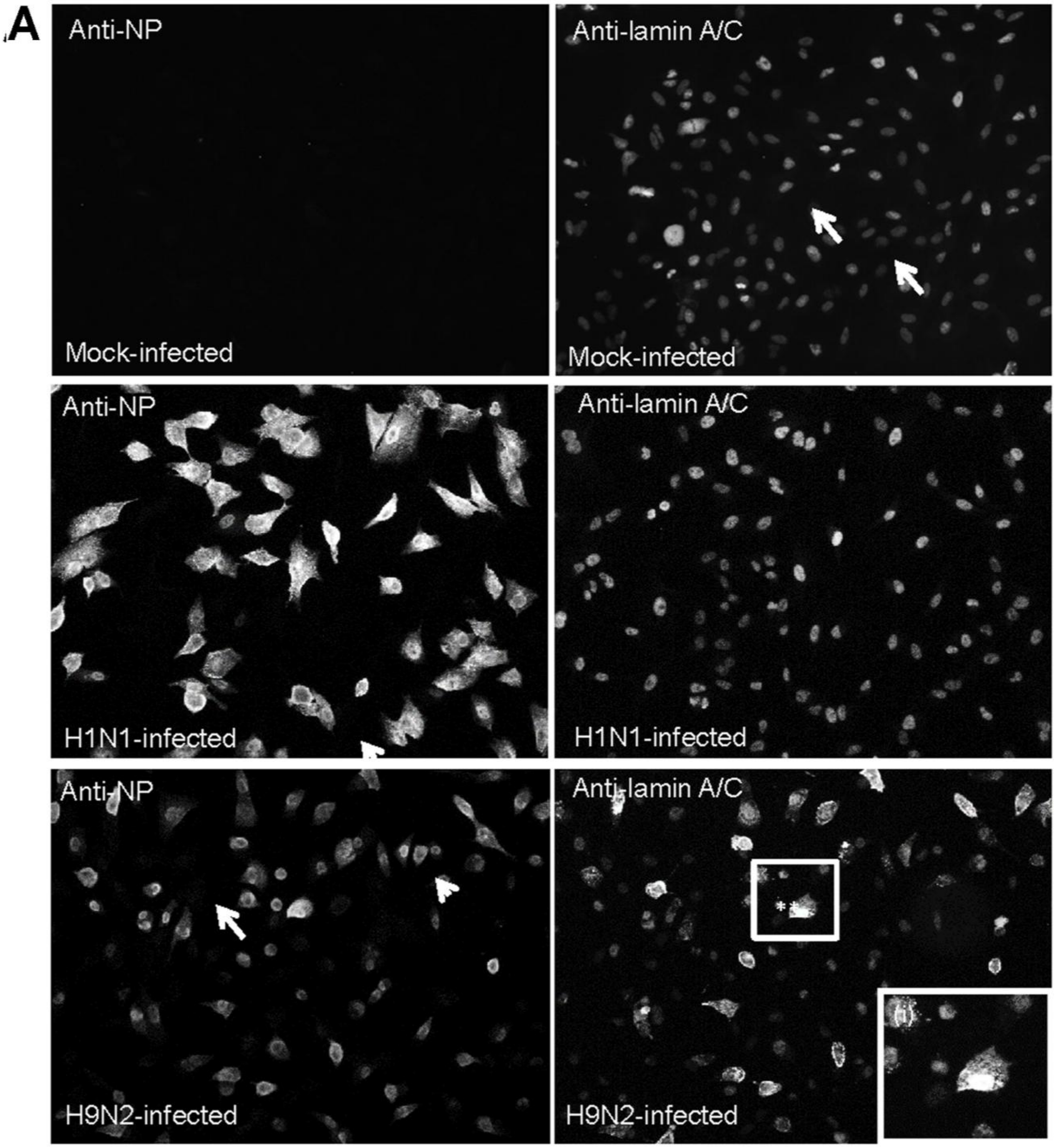
A
Anti-NP
Mock-infected
Anti-lamin A/C
Mock-infected
Anti-NP
H1N1-infected
Anti-lamin A/C
H1N1-infected
Anti-NP
H9N2-infected
Anti-lamin A/C
H9N2-infected

B

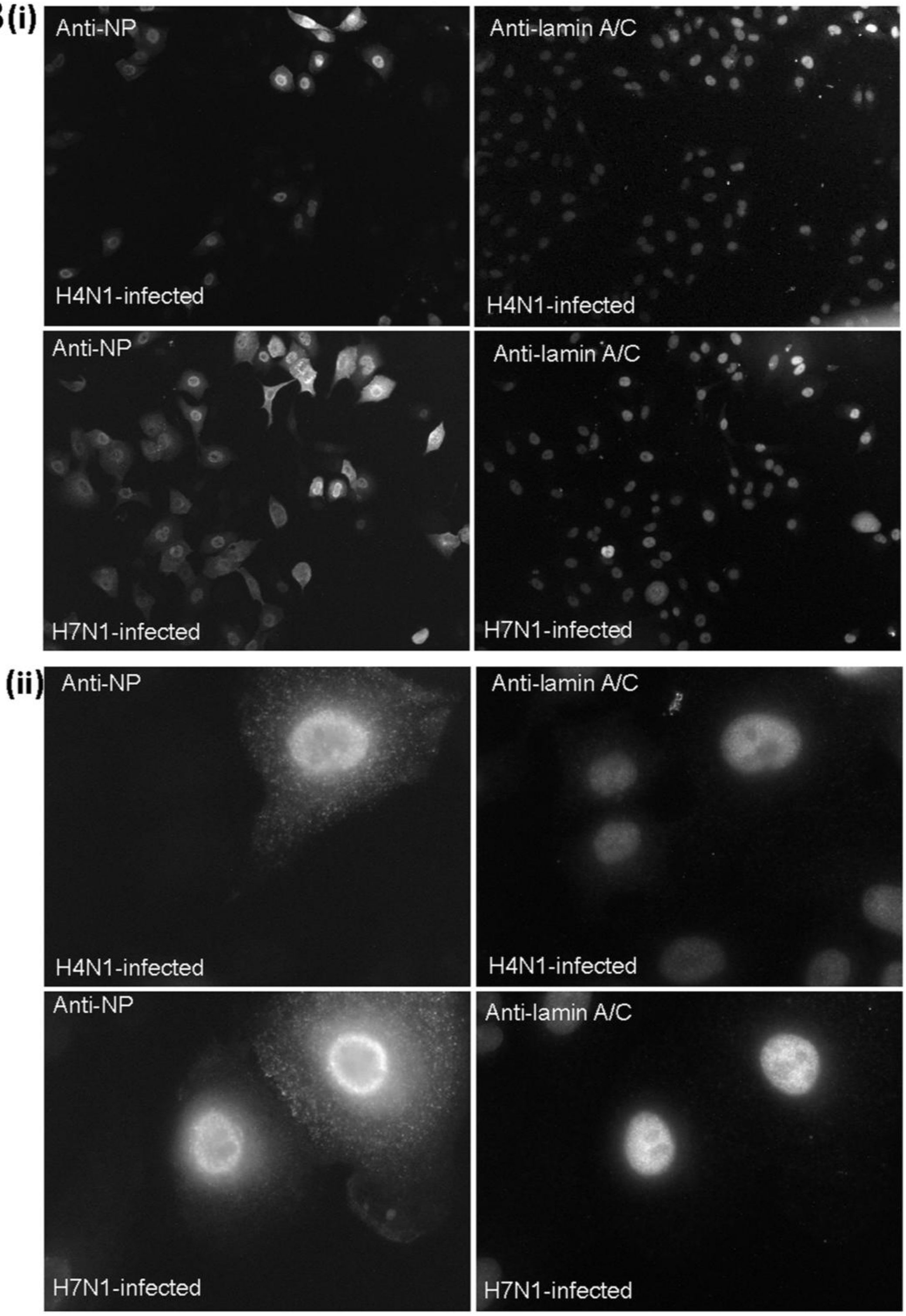
(i)
Anti-NP
H4N1-infected
Anti-lamin A/C
H4N1-infected
Anti-NP
H7N1-infected
Anti-lamin A/C
H7N1-infected
(ii)
Anti-NP
H4N1-infected
Anti-lamin A/C
H4N1-infected
Anti-NP
H7N1-infected
Anti-lamin A/C
H7N1-infected

C

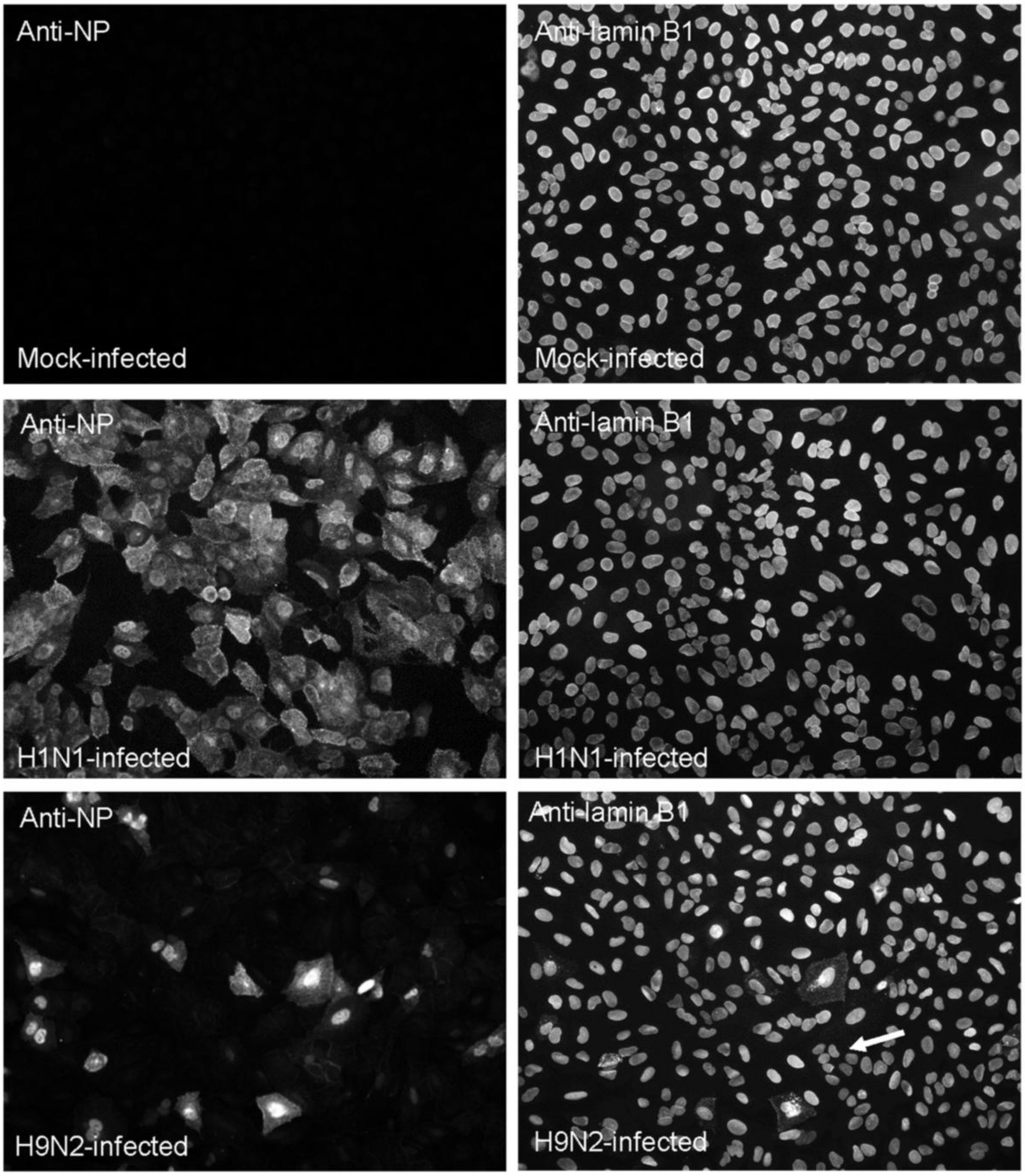
Anti-NP
Mock-infected
Anti-lamin B1
Mock-infected
Anti-NP
H1N1-infected
Anti-lamin B1
H1N1-infected
Anti-NP
H9N2-infected
Anti-lamin B1
H9N2-infected

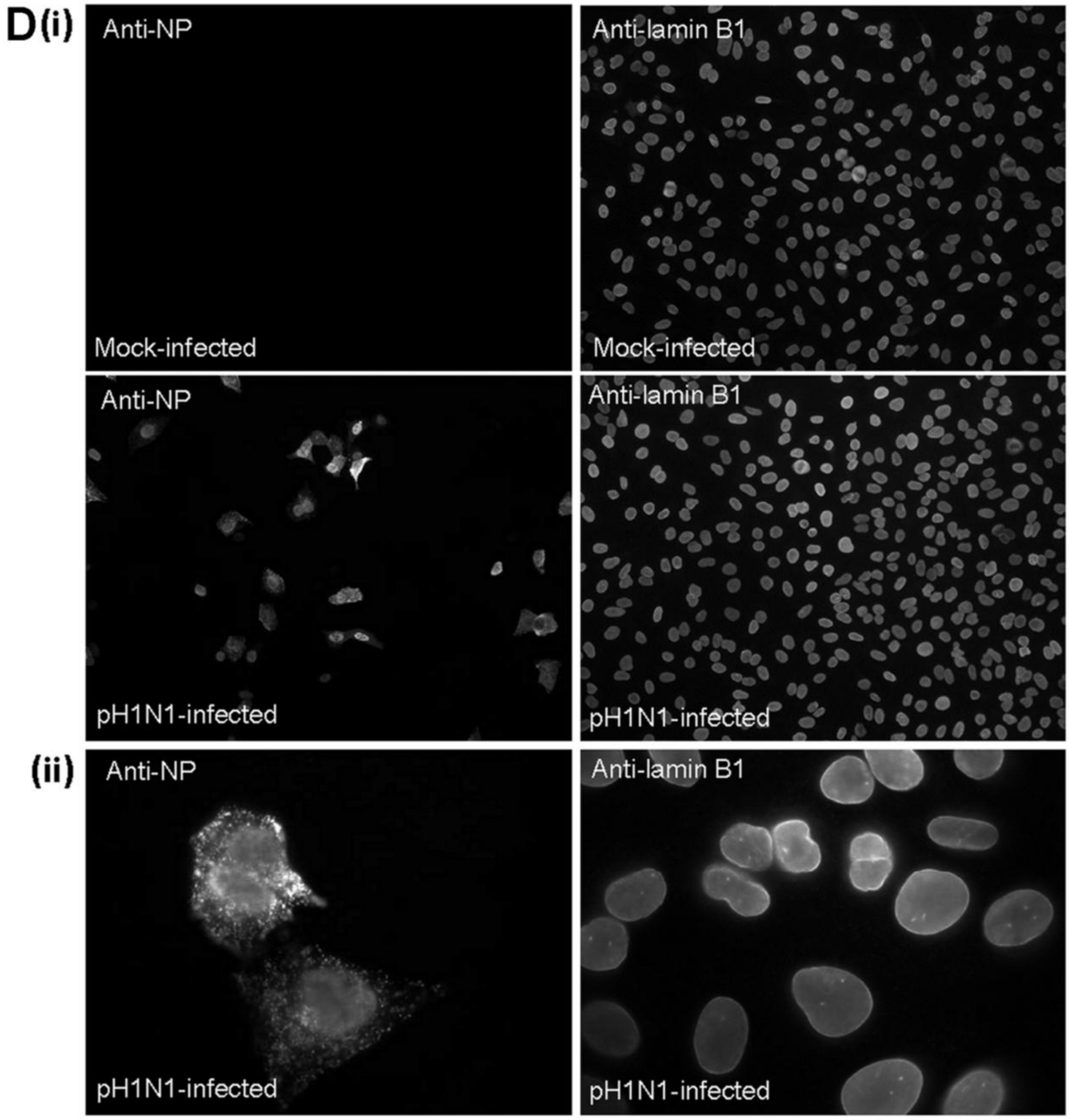
D(i)
Anti-NP
Mock-infected
Anti-lamin B1
Mock-infected
Anti-NP
pH1N1-infected
Anti-lamin B1
pH1N1-infected
(ii)
Anti-NP
pH1N1-infected
Anti-lamin B1
pH1N1-infected

SFig. 6

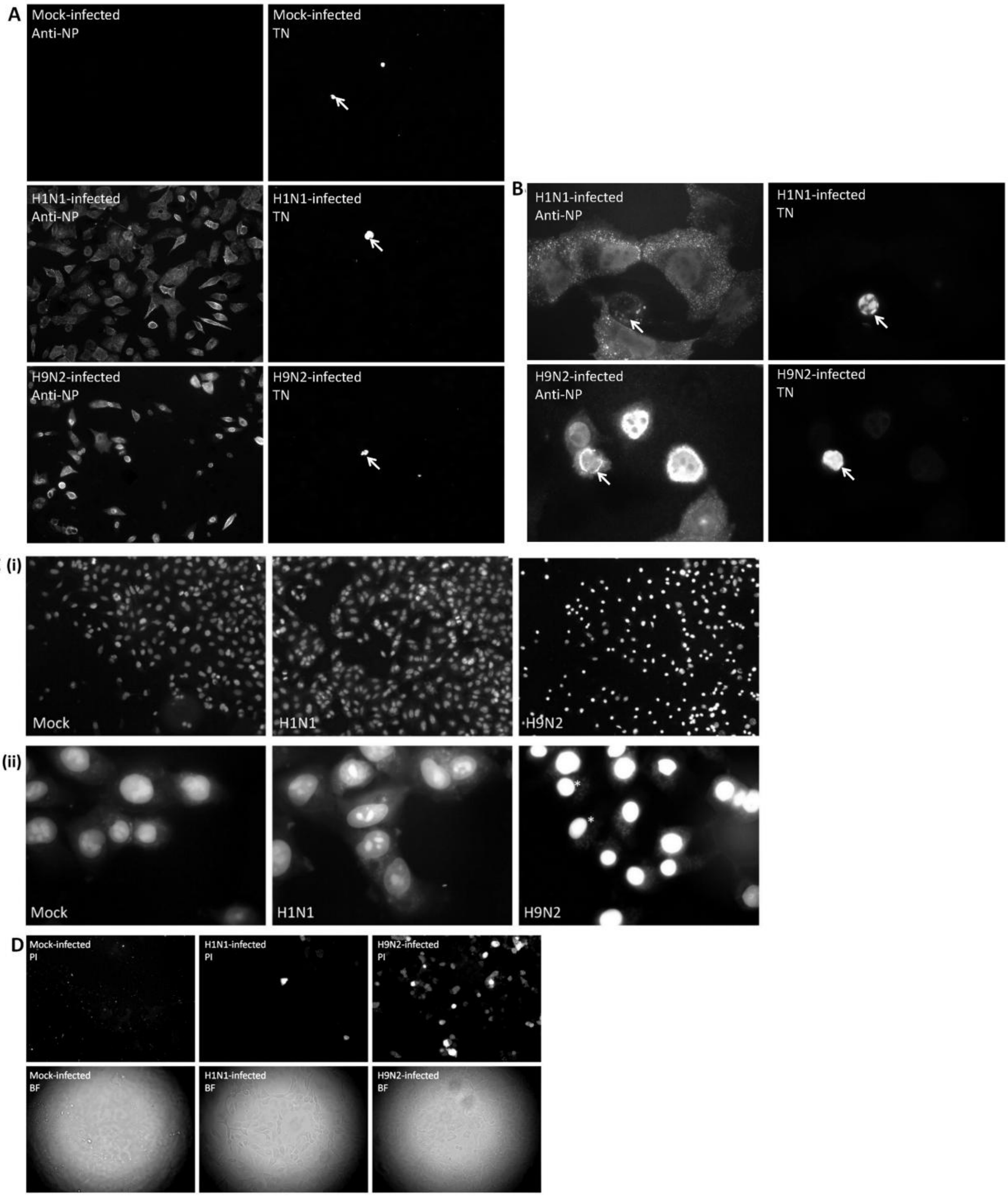
A
Mock-infected
Anti-NP
Mock-infected
TN
H1N1-infected
Anti-NP
H1N1-infected
TN
H9N2-infected
Anti-NP
H9N2-infected
TN
B
H1N1-infected
Anti-NP
H1N1-infected
TN
H9N2-infected
Anti-NP
H9N2-infected
TN
C (i)
Mock
H1N1
H9N2
(ii)
Mock
H1N1
H9N2
D
Mock-infected
PI
H1N1-infected
PI
H9N2-infected
PI
Mock-infected
BF
H1N1-infected
BF
H9N2-infected
BF

SFig. 7

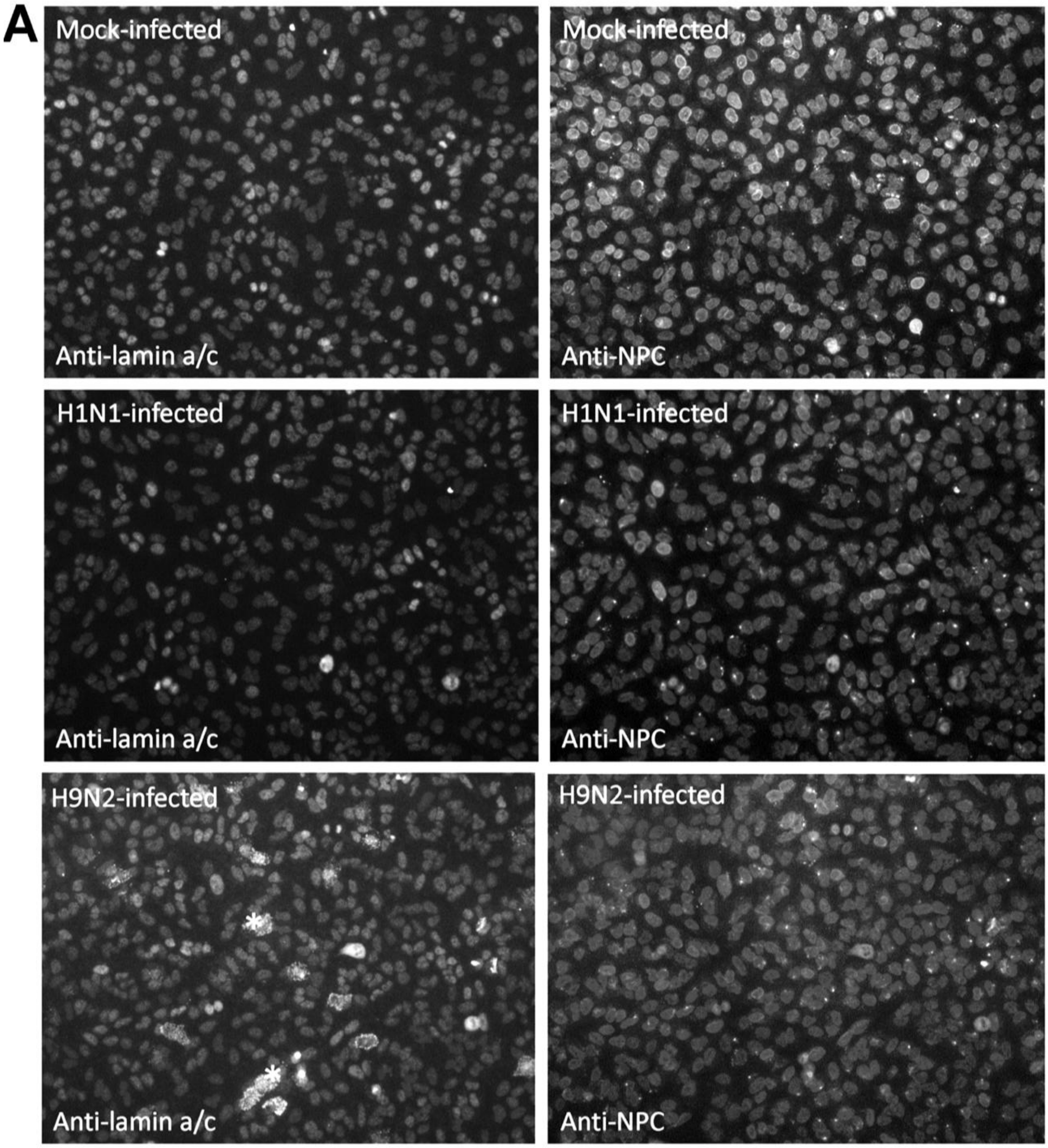
A
Mock-infected
Anti-lamin a/c
Mock-infected
Anti-NPC
H1N1-infected
Anti-lamin a/c
H1N1-infected
Anti-NPC
H9N2-infected
Anti-lamin a/c
H9N2-infected
Anti-NPC

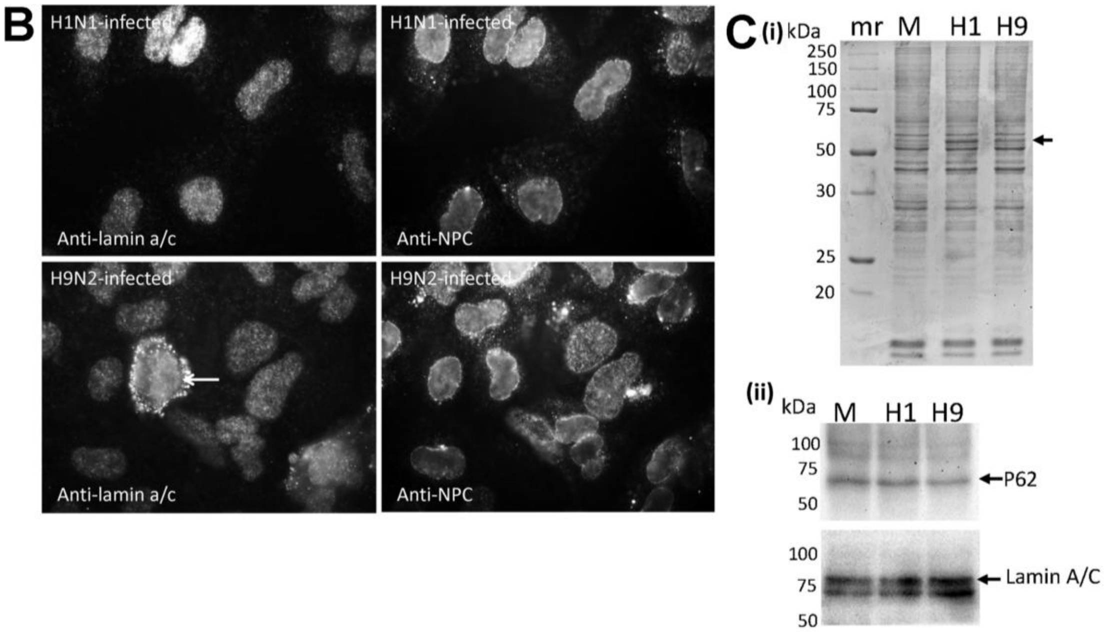
B
H1N1-infected
Anti-lamin a/c
H1N1-infected
Anti-NPC
H9N2-infected
Anti-lamin a/c
H9N2-infected
Anti-NPC
C (i) kDa
mr M H1 H9
250
150
100
75
50
30
25
20
(ii) kDa M H1 H9
100
75
50
P62
100
75
50
Lamin A/C

Stable 1

a

H1N1/WSN virus genes	Primers	Sequence (5'-3')
NP	NP-EcoRI-FW	CCGGAATTCCGGAGCAAAAGCAGGGTA
	NP-KpnI-RV	CGGGGTACCCCGAGTAGAAACAAGGGTATTTTT
PA	PA-KpnI-FW	CGGGGTACCCCGAGCGAAAGCAGGTAC
	PA-XhoI-RV	CCGCTCGAGCGGAGTAGAAACAAGGTACTT
PB1	PB1-KpnI-FW	CGGGGTACCCCGAGCGAAAGCAGGCA
	PB1-XhoI-RV	CCGCTCGAGCGGAGTAGAAACAAGGCATTT
PB2	PB2-KpnI-FW	CGGGGTACCCCG AGCGAAAGCAGGTC
	PB2-XhoI-RV	CCGCTCGAGCGGAGTAGAAACAAGGTCGTTT

pH1N1/471 virus genes	Primers	Sequence (5'-3')
NP	NP-EcoRI-FW	CCGGAATTCCGGAGCAAAAGCAGGGTA
	NP-KpnI-RV	CGGGGTACCCCGAGTAGAAACAAGGGTATTTTT
PA	PA-KpnI-FW	CGGGGTACCCCGAGCGAAAGCAGGTAC
	PA-XhoI-RV	CCGCTCGAGCGGAGTAGAAACAAGGTACTT
PB1	PB1-KpnI-FW	CGGGGTACCCCGAGCGAAAGCAGGCA
	PB1-XhoI-RV	CCGCTCGAGCGGAGTAGAAACAAGGCATTT
PB2	PB2-XhoI-FW	CCGCTCGAGCGGAGCGAAAGCAGGTC
	PB2-XhoI-RV	CCGCTCGAGCGGAGTAGAAACAAGGTCGTTT

H9N2 virus genes	Primers	Sequence (5'-3')
NP	NP-EcoRI-FW	CCGGAATTCCGGAGCAAAAGCAGGGTA
	NP-KpnI-RV	CGGGGTACCCCGAGTAGAAACAAGGGTATTTTT
PA	PA-BglII-FW	GGAAGATCTTCCAGCGAAAGCAGGTAC
	PA-BglII-RV	GGAAGATCTTCCAGTAGAAACAAGGTACTT
PB1	PB1-KpnI-FW	CGGGGTACCCCGAGCGAAAGCAGGCA
	PB1-XhoI-RV	CCGCTCGAGCGGAGTAGAAACAAGGCATTT
PB2	PB2-XhoI-FW	CCGCTCGAGCGGAGCGAAAGCAGGTC
	PB2-XhoI-RV	CCGCTCGAGCGGAGTAGAAACAAGGTCGTTT

b

Virus	Gene	Sequence (5'-3')	Probe Sequence (UPL Probe #)
H1N1	NP FW	TGGAATCAAGTACCCTTGAACTG	TCTGGTCC (#93)
	NP RV	GCCCTCTGTTGATTGGTGTT	
	PA FW	CGGAAAAGGCAATGAAAGAG	TCCTCTCC (#55)
	PA RV	CTGCAAATTTGTTTGTTTCGAT	
	PB1 FW	GCTCCAATAATGTTCTCAAACAAA	ACTGGGAA (48)
	PB1 RV	TCTTGCTCAAACATGTACCC	
	PB2 FW	TCCGCAGTTCTGAGAGGATT	CTGGGCAA (61)
	PB2 RV	TGCTGGTCCATACTCCTGTC	

Virus	Gene	Sequence (5'-3')	Probe Sequence (UPL Probe #)
H9N2	NP FW	CCCGAAGAAAACTGGAGGTC	GAAGGAGG (134)
	NP RV	TCAGCTCTCTCATCCATTTCC	
	PA FW	AAAGCGGACTACACCCTTGA	GAGAGCAG (108)
	PA RV	AGTGAACAGCCTGGTTTTGATT	
	PB1 FW	CTTGAAGTGGGAATTGATGGA	CCAGGGCA (37)
	PB1 RV	ATGGGTTCTGAGGATTGCAC	
	PB2 FW	ACAAAAACCACTGTGGACCAT	CTGCTCTC (108)
	PB2 RV	CATTGCCATCATCCATTTCA	

Host	Gene	Sequence (5'-3')	Probe Sequence (UPL Probe #)
Human	EF FW	TGGGAGGGTTGCTTTGATTA	ACACTGGA (#47)
	EF RV	TGACAGGCAATCAGCAACAT	

SFigure legends.

SFig. 1. Distribution of the RNP and chromatin in the nucleus of H1N1 and H9N2 virus infected cells. A549 cells were infected with either H1N1/WSN(H1N1) or H9N2 viruses using a multiplicity of infection (MOI) of 5 and at 16 hrs post-infection (hpi) the cells were stained using anti-NP and anti-histone H4 (H4) and **(A)** imaged by fluorescence microscopy (objective x20 magnification). The nuclear staining is highlighted (white arrows). **(B and C)** Individual anti-NP and anti-histone H4 (H4) co-stained **(B)** H1N1/WSN and **(C)** H9N2 virus were examined using confocal microscopy. (i) Images at the same focal plane ae shown. (ii) Enlarged image taken from the nucleus of co-stained cells are shown. The cytoplasmic anti-NP staining in H1N1 virus-infected cells are highlighted (white arrow heads).

SFig. 2. **Distribution of the NP in virus-infected A549 cells.** A549 cells were mock-infected or infected with either H1N1/WSN(H1N1), H9N2, H4N1, H7N1 or pH1N1/471 (pH1N1) using a multiplicity of infection (MOI) of 5. At 16 hrs post-infection (hpi) the cells were stained using anti-NP and **(A)** imaged by fluorescence microscopy using identical camera settings (objective x20 magnification). Insert showing enlarge image of representative cells imaged in pH1N1 virus infected cells. **(B)** Cells imaged by fluorescence microscopy using identical camera settings (objective x100 magnification). The cytoplasmic NP staining (*) and nucleus-specific NP staining (white arrows) are indicated.

SFig. 3. **Expression of the recombinant polymerase genes of the H1N1/WSN(H1N1), pH1N1/471 (pH1N1) or H9N2(H9N2) viruses.** HEK293T cells were either transfected with pCAGGS or with the pCAGGS containing the **(A)** NP, **(B)** PA, **(C)** PB1 and **(D)** PB2 genes of the H1N1/WSN(H1N1), pH1N1/471 (pH1N1) or H9N2(H9N2) viruses. After 24 hrs post-transfection the cells were stained using **(A)** anti-NP **(B)** anti-PA, **(C)** anti-PB1 and **(D)** anti-

PB2 and imaged by fluorescence microscopy (objective x20 magnification) using identical camera settings for each antibody combination.

SFig. 4. PA protein sequences. (A) Sequence comparison of the PA protein of the viruses used in this study. The amino acid residues unique to the H9N2 sequence are highlighted in yellow. **(B)** The amino acids associated with human (green) and avian (red) host adaptation as proposed by Miotto et al 2010.

SFig. 5. Lamin A/C and B1 distribution in influenza virus-infected cells. A549 cells were **(A)** mock-infected or infected with the H1N1 and H9N2 viruses and **(B)** H4N1 and H7N1 viruses and at 20 hrs post-infection (hpi) the cells were co-stained using anti-NP and anti-lamin A/C. The stained cells were imaged by fluorescence microscopy **(A and B(i))** (objective x20 magnification) or **(B(ii)) (**objective x100 magnification). Inset in **(A)** is enlarged image of area highlighted (white box). **(C)** A549 cells were mock-infected or infected the H1N1 and H9N2 viruses and **(D)** pH1N1 viruses and at 20 hpi the cells were co-stained using anti-NP and anti-lamin B1. The stained cells were imaged by fluorescence microscopy using identical camera settings at **(C and D(i))** (objective x20 magnification) and **(D(ii))** (objective x100 magnification). The anti-lamin B1 stained nuclei (white arrow) are highlighted.

SFig. 6. Increased apoptosis is not observed in H9N2 virus-infected A549 cells. (A) A549 cells were mock-infected or infected with the H1N1 and H9N2 viruses and at 20 hrs post-infection (hpi) the cells were co-stained using TUNNEL staining (TN) and anti-NP. The stained cells were imaged by fluorescence microscopy (objective x20 magnification) using identical camera settings. The sporadic TN-stained cells (white arrows) are highlighted. **(B)** TN and anti-NP co-stained cells were imaged by fluorescence microscopy (objective x100

magnification) using identical camera settings. TN-stained cells (white arrows) are highlighted. **(C)** A549 cells were mock-infected or infected with the H1N1 and H9N2 viruses and at 20 hpi the cells were permeabilised and stained using propidium iodide (PI) and the cell viewed by fluorescence microscopy using (i) (objective x20 magnification) and (ii) (objective x100 magnification). The intensely PI-stained nuclei are highlighted (*). **(D)** A549 cells were mock-infected or infected with the H1N1 and H9N2 viruses and at 18 hpi the live cells were stained using PI and imaged using IF microscopy in fluorescence-mode (PI) and bright-field-mode (BF) (objective x20 magnification) using identical camera settings.

SFig. 7 Anti-NPC staining in influenza virus-infected cells. (A) A549 cells were mock-infected or infected with the H1N1 and H9N2 viruses and at 20 hrs post-infection (hpi) the cells were co-stained using anti-lamin A/C and anti-NPC. The stained cells were imaged by fluorescence microscopy (objective x20 magnification) using identical camera settings. The cytoplasmic anti-lamin A/C staining (*) is highlighted. **(B)** anti-lamin A/C and anti-NPC co-stained H1N1 and H9N2 virus-infected cells were imaged by fluorescence microscopy (objective x100 magnification) using identical camera settings. The cytoplasmic anti-lamin A/C staining (white arrow) is highlighted. **(C)** Nuclei were prepared from mock-infected cells (M) and cells infected with H1N1/WSN (H1), and H9N2 (H9) viruses at 24 hpi. (i) Commassie Brilliant Blue-stained polyacrylamide gel of the nuclei preparations is shown. The molecular mass marker lane (mr) and the position of the NP (black arrow) are highlighted. (ii) The nuclei preparations were analysed by immunoblotting using anti-NPC (p62) and anti-lamin A/C and protein species of the expected sizes are indicated.

Stable 1. List of virus-specific primers used in this study. (a) List of primer sequences: forward (FW) and reverse (RV) primers for cloning of NP, PA, PB1 and PB2 genes of

H1N1/WSN, pH1N1/471 and H9N2 strains into the pCAGGS cloning vector. The underlined nucleotides represent the restriction enzyme. (b). List of primers used for qPCR analysis. List of primer sequences and UPL probes (Roche) designed for real-time qPCR of influenza H1N1/WSN and H9N2 gene segments. Primer sequences and UPL probes (Roche) used for real-time qPCR of human host gene is also shown.
